# A novel optimal design approach for bladeless wind turbines considering mechanical properties of composite materials used

**DOI:** 10.1038/s41598-024-82385-9

**Published:** 2025-01-08

**Authors:** Zeinab Mohamed, Moataz Soliman, Mohamed Feteha, E. Saber

**Affiliations:** 1 Technologies and Materials of Renewable Energy Program, Department of Materials Science, Institute of Graduate Studies and Research, Alexandria University, Alexandria, Egypt; 2 Department of Materials Science, Institute of Graduate Studies and Research, Alexandria University, Alexandria, Egypt; 3 College of Engineering, Arab Academy for Science, Technology and Maritime Transport, Alexandria, Egypt

**Keywords:** Wind energy, Bladeless wind turbine, Energy harvesting, Vortex-induced vibration, Mechanical engineering, Energy harvesting, Devices for energy harvesting

## Abstract

Bladeless wind turbines face operational limitations due to the lock-in phenomenon. This study introduces two novel mechanisms for designing bladeless wind turbines to address this issue, enabling operation across a broad wind speed range from 2 to 10 m/s while ensuring that lock-in conditions are satisfied at any wind speed within this range. The study aims to maintain optimal performance without any decline that is observed in conventional bladeless wind turbines by controlling the turbine’s natural frequency through implementing these mechanisms, either by adjusting the effective length of the stand or by incorporating an additional mass in the hollow mast, or both. A mathematical model including dynamic analysis is constructed to adjust natural frequency to match the shedding frequency at the specified wind speed. Validation of our model shows high accuracy. Numerical results demonstrate that applying these mechanisms ensures the turbine is optimally designed across varying parameters. Findings reveal that for lower flexural modulus values, the first mechanism alone can achieve a 99.2% increase in mechanical efficiency at 7 m/s. For higher flexural modulus values, incorporating the second mechanism is essential to reduce the turbine’s overall size. This integrated approach improves efficiency with a 55.7% increase.

## Introduction

Conventional wind turbines, which are essential for capturing wind energy efficiently, have inherent drawbacks such as noise pollution, visual and environmental impacts, efficient operation only at high wind speeds, high initial costs, intermittent power, regular maintenance, and construction limitations in urban areas has driven the development of alternative technologies to address these issues. In recent years, researchers have explored more sustainable method for harvesting wind energy through vortex-induced vibration (VIV). The flow-induced vibration harvesters are one type of novel wind energy harvesting approach that is based on the aero-elasticity interaction phenomena^1^]. As blunt bodies are immersed in a continuous, uniform fluid flow, the flow field near their surfaces can sometimes generate shedding vortices. In flexible mounting bodies, vortex-induced vibrations (VIV) are caused by the periodic shedding of vortices in conjunction their surfaces, which causes a fluctuating pressure field around the body^2–6^. Using the VIV thought in wind energy harvesters has led to an abundance of research investigations, particularly for micro or miniature-size harvesters^7^. The basic concept was simple: a flexibly positioned horizontal cylinder free to move normal to the free-stream and connected to a linear electromagnetic alternator. This and comparable structures, however, are difficult to scale up economically due to their complex basis of support^8,9^. A vertical alternative of the harvester, known as vortex-induced vibration bladeless wind turbine (VIV-BWT), was designed to reduce the requirement for complex-supporting structures^10^. It is made included of a cylindrical body (mast) that stands vertically at the end of a flexible cantilever. An electromagnetic or piezoelectric PTO is frequently used to convert induced mechanical power to electric power. The major characteristics of the harvesters are their basic structure, lack of moving components and minimal maintenance needs. The VIV-harvester’s power output is confined to a specific region where the vortex shedding frequency is near to the structural natural frequency; which is known as the lock-in phenomenon^11^. Surprisingly, under the lock-in situation, synchronization occurs between structural oscillation and vortex shedding, when body motion acts as a magnification, organizer, and synchronizer of the phenomenon^12^. The collected energy drops considerably if the incoming wind speed is outside the zone corresponding to the lock-in phenomenon^13^. Although several studies have documented a drop in VIV power generation and efficiency outside the lock-in zone, only a small number of studies have offered a solution to this problem. There are four types of efficiency-boosting solutions for VIV energy harvesters: (1) modifying the geometric shape and orientation of the blunt body^14–20^, (2) optimizing the electric circuit and control algorithm^21–25^, (3) using nonlinear flexible support structures^26,27^, and (4) utilizing multiple sources of kinetic energy^28,29^.

As previously stated, the lock-in phenomena is limited to a small range near the structural natural frequency. A few studies went so far as to employ a tuning mechanism that altered the structural natural frequency and so improve output performance. Sun and Seok^30^ suggested a VIV piezoelectric energy harvester that self-tunes. It is made up of a circular cylinder-shaped slidable bluff body. The balance of drag and centrifugal forces causes self-adjustment of the effective beam length and, as a result, the system’s resonances. The results show that a broadband lock-in condition of about 1–2.7 m/s is achievable. However, such arrangements require that the harvester structure is fixed horizontally along the wind speed direction. Zhang et al.^27^ presented a magnetic repulsive flux-based supporting structure for a VIV harvester with nonlinear restoring force. It has been found that the distance between each of the repulsive magnets greatly alters the harvester’s inherent frequency. In comparison to traditional harvesters, the synchronization region and output power have grown by 138% and 29%, respectively. Similar results were obtained in Naseer et al.^31^, where the synchronization zone was expanded by utilizing two attractive magnets instead, one mounted along the mast side and the other fixed. Yazdi^32^ presented a tuned-mass mechanism for BWT in conjunction with model predictive control (MPC) to expand the wind speed spectrum across which the turbine is effective. The MPC is designed to determine the best control inputs for the tuned mass actuator and piezoelectric harvester. When compared to the traditional BWT without tuning (1.01–1.1 m/s), a broader effective wind speed band of (1.05–1.92 m/s) is obtained. However, at greater wind speeds, performance is still far from optimal (efficiency is less than 5% at 2.6 m/s). However, at small-to-medium size VIV-BWT (2–5 m mast length), the solutions proposed for enhancing performance are inefficient, especially at wind speeds exceeding 3 m/s. The work given by Magdy et al.^33^ provides an innovative semi-active method for modifying the turbine response to increase the effective power output zone. A supporting mechanism is proposed for changing the elastic member length and, as a result, the structural natural frequency. The suggested tuning approach then attempts to keep the effective parameters at their ideal power production value. It was discovered that the proposed method significantly expanded the effective wind speed range. Over a wind speed range of 3.3–6 m/s, the conversion efficiency is more than or equivalent to 15%. Bahadur^34^ examined the dynamics of a tunable electromagnetic vortex bladeless wind turbine. This is made up of a progressive-rate spring connected to an oscillating magnet inside an electromagnetic coil. As the wind speed changes, the spring stiffness is gradually modified to match the shedding frequency. He discovered that as compared to a traditional bladeless turbine, the output power of the 2 m long adjustable turbine is tens of times better. He gave an example, stating that the output RMS power of the tunable turbine is around 1105 mW at a wind speed of 4.22 m/s, compared to 17 mW for the standard BWT.

In addition to focusing on the possibilities of tuning mechanisms to improve bladeless wind turbine performance, the choice of appropriate materials for the turbine’s flexible element is critical to ensuring satisfactory performance and strength as they affect the overall stiffness of the structure, consequently influencing its natural frequency, which affects all BWT performance^35,36^. Carbon and glass fibers were found to be the best materials for fabricating the main components of bladeless wind turbines^37^. Furthermore, composite materials mechanical properties can be controlled by changing their fabrication parameters, such as the number of layers and their orientation^38^. This allows for tailoring the strength, stiffness, and other mechanical characteristics to meet specific requirements for different applications^39^. The combination of high strength, stiffness, damage tolerance and the ability to tailor properties through fiber orientation and layup make carbon fiber composites an ideal choice for applications that require exceptional fatigue resistance, such as aerospace, automotive and wind energy^41^.

**Fig. 1:**
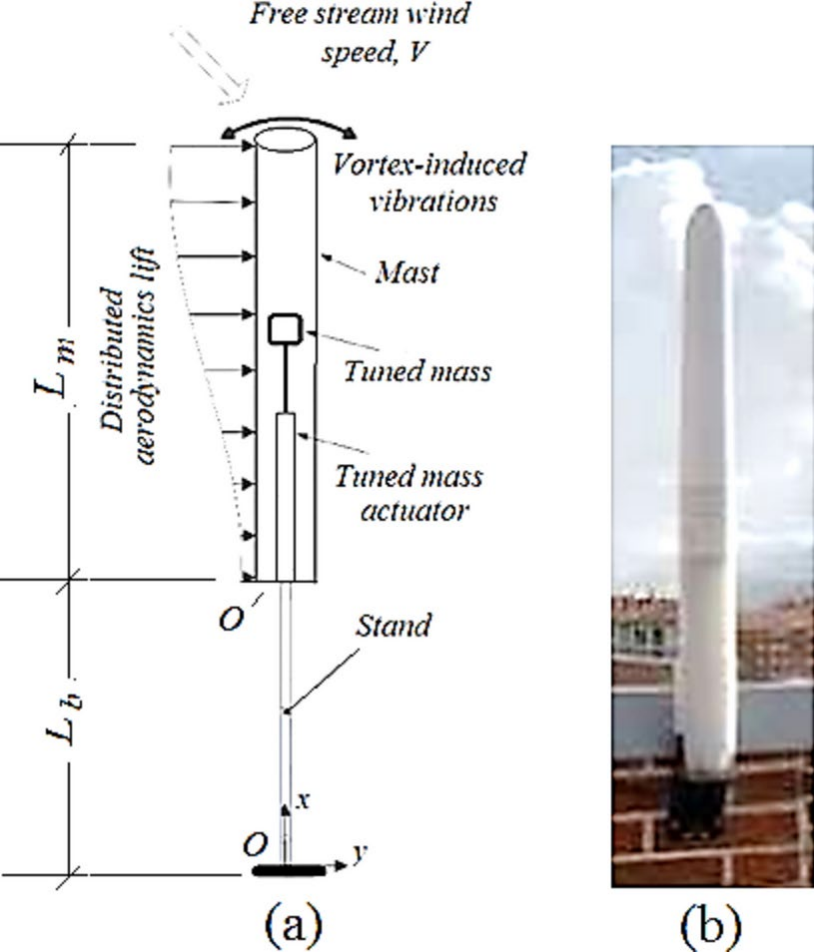
(**a**) Schematic drawing of the proposed VIV.BWT. (**b**) A real shape of the BWT.

The current study aims to provide an approach for designing for bladeless wind turbine, allowing it to operate over a wide wind speed range of 2–10 m/s while maintaining the lock-in condition at any wind speed within this range Addressing the issue of performance loss at high wind speeds due to a mismatch between vortex shedding and natural frequencies, the authors propose two tuning mechanisms: the elastic tuning mechanism, which modifies the elastic stand’s effective length, and the tuned mass mechanism (TMM), as shown in Fig. 1a, which moves the additional tuning mass inside the hollow mast, which change the fundamental mass of VIV and hence change its inherent frequency. These mechanisms ensure the turbine’s resonance adapts to varying wind speeds, enhancing its performance and energy harvesting potential.

The novelty of this study lies in the integration of the tuning mass mechanism and the elastic tuning mechanism that has not been examined in previous studies. This integration requires a novel mathematical treatment and modeling. The use of the equivalent mass and equivalent mass moment of inertia of the mast unit at the tip of the stands enables an analysis of the turbine’s characteristic equation, Incorporating the turbine design parameters and the geometric parameters of the stand and mast.

The article is organized as follows. In section "Materials and methods" the system configuration and its theoretical model for the two proposed approaches are presented, as well as the dynamic response and key performance metrics. Section "Numerical Simulation and Model Validation" describes the numerical simulation and model validation. In Section "Results and discussion", the results and discussion are presented, detailing the impact of both approaches and their integration on the dynamic response and performance of BWT. Section "Limitations" addresses the limitations and challenges of this study. Key findings and suggestions for future research are presented in section "Conclusions".

## Materials and methods

### System description

The current configuration of the BWT is presented in Fig. 1a, featuring a flexible stand beam and a stiff mast assembly. The mast unit comprises a mast, tunable mass, and a guiding rod, which represent the tuned mass mechanism. The flexible beam that allows the BWT to vibrate is constructed from a carbon fiber composite material. Table 1 lists the mechanical properties of carbon fiber composite materials used in this study.

**Table 1:** Mechanical properties of carbon fiber/epoxy laminated composites^39^.

Sample code	Resin type	Number of laminates	Fiber orientation (°)	Tensile strength (MPa)	Tensile modulus (GPa)	Flexural strength (MPa)	Flexural modulus (GPa)
LY3/1	LY 5052	3	0, 90, 0	329	27.5	525	28.5
LY3/2	LY 5052	3	0, 35, 0	1017	73.4	1110	51.9
LY3/3	LY 5052	3	90, 45, 90	20	6.8	140	4.1
EM3/3	EM 500	3	90, 45, 90	21.4	6.3	85.4	2.45
EP5/4	EPON 828	5	0, − 35, 0, + 35, 0	647	71.58	911	57.57

Composites were manufactured by hand-lay-up process, using a fiber-to-resin ratio of 40:60 (w:w).

### Theoretical model

The turbine’s geometric characteristics are presented in Table 2. The flexible beam allows the BWT to vibrate, while the mast causes vortex shedding and provides an oscillating aerodynamic force. Because the stand is tiny in comparison to the mast, the aerodynamic force on it may be ignored.

**Table 2:** Specifications of the proposed VIV_-_BWT system_._

_Parameter_	_Description_	_Value_	_Schematic representation of the planned VIV-BWT_
$D_{m}$	_Mast diameter_	_200–500 mm_	
$L_{m}$	_Mast length_	_5 m_	
$t_{m}$	_Mast thickness_	_0.7 mm_	
$\rho_{m}$	_Mast density_	$1500\,kg/m^{3}$	
$D_{b}$	_Stand (Beam) diameter_	_*60 mm*_	
$t_{b}$	_Stand thickness_	_*0.4 mm*_	
$L_{b}$ $L_{b\, - \,eff}$	_Stand length_ _Effective stand length_	_*Required*_	
$\rho_{b}$	_Stand density_	$1500\,kg/m^{3}$	
$E$	_Flexural modulus of the stand [39]_	$2.45 - 51.9\,\,GPa$	
$L_{rod}$	_Guide rod length_	$0.9\,L_{m}$	
$D_{ro}$	_Outside diameter of the guide rod_	$30\,mm$	
$D_{ri}$	_Inside diameter of the guide rod_	$0.28\,mm$	
$\rho_{rod}$	_Density of the rod (Alum)_	$2700\,kg/m^{3}$	
$t_{rb}$	_Thickness of the guide rod base_	$50\,mm$	
$D_{rb}$	_Diameter of the guide rod base_	$D_{m} - 2\,t_{m}$	
$\rho_{rb}$	_Density of the rod base (Alum)_	$2700\,kg/m^{3}$	
$m_{t}$	_Tuned mass_	$2.5 - 10\,\,kg$	
$D_{to}$	_Outer diameter of the tuned mass_	$100\,mm$	
$D_{ti}$	_Inner diameter of the tuned mass_	$D_{ti} = D_{ro}$	
$\rho_{tm}$	_Density of the tuned mass (steel)_	$7850kg/m^{3}$	

**Fig. 2:**
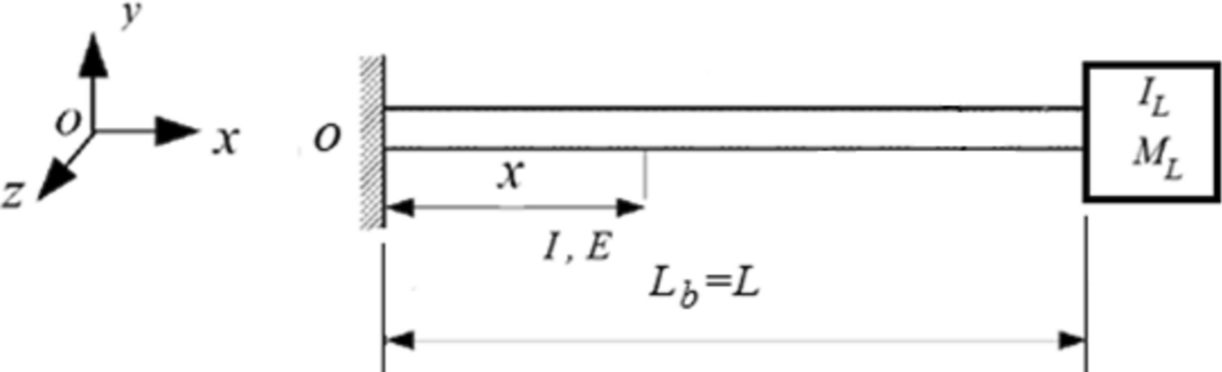
A sketch of the cantilever beam with a tip mass.

The suggested turbine configuration may be represented mathematically as a cantilevered uniform beam with a tip mass attachment ($M_{L} \,\,and\,\,I_{L}$) as shown in Fig. 2. $M_{L}$ is the equivalent mass of the mast unit at beam tip and $I_{L}$ is the mast mass moment of inertia at $x = L_{b}$, where $L_{b}$ is the length of the working cantilever beam. The first moment of mass about the point "O" may be used to obtain equivalent mass of the mast unit at the tip of the beam, $M_{L}$, which can be expressed as.

1 $$M_{L} = m_{m} + m_{rod} + m_{t} \, + \frac{1}{{L_{b} }}\,(m_{m} \,\overline{L}_{m} + m_{rod} \,\overline{L}_{rod} \, + m_{t} \,s)$$

where $m_{m}$ is the mass of the mast, $m_{rod}$ is the mass of the guide rod, $m_{t}$ is the tunable mass. The distances $\overline{L}_{m} \,,\,\,\overline{L}_{rod} \,\,and\,\,s\,$ are between the tip of the beam (stand) to the centers of the mast, guide rod, and tunable mass, respectively. The parallel-axes theorem has been used in determining mass moment of inertia about the tip of the beam, at point $O^{\prime}$. The mass moment of inertia $I_{L}$ about z-axis is given by,

2 $$I_{L} = \left( {I_{m} + m_{m} \,\overline{L}_{m}^{\,2} } \right) + \left( {I_{rod} \,\overline{L}_{rod}^{\,2} } \right) + \left( {I{}_{t} + m_{t} \,s^{2} } \right)$$

Thus, the in order to investigate the influence of the mast unit elements on the structure’s natural frequency to achieve resonance, the turbine represented as uniform cantilever beam with a tip mass.

#### Natural frequencies of a cantilever beam with an end mass $(M_{L} \,\,and\,\,I_{L} )$

The governing equation of motion for undamped free vibrations of a uniform Euler–Bernoulli beam may be written as^49^:

3 $$\frac{{\partial^{4} }}{{\partial \,x^{4} }}\left( {E\,I\,Y(x,t)} \right) + m_{s} \,\frac{{\partial^{2} Y(x,t)}}{{\partial \,t^{2} }} = 0$$

where, $E$ is the Flexural modulus of elastic beam, $I$ is the second moment of area of the cross section, and $m_{s}$ is the mass per unit length of the stand (beam). To solve Eq. (3), which is fourth-order in geographical variables and second-order in time, the variable separation approach is to be applied as follows:

4 $$Y(x,t) = \sum {\varphi_{i} \,(x)\,y_{i} \,(t)}$$

where $\varphi_{i} \,(x)$ and $y_{i} \,(t)$ are called the mode shape and the modal response factor, respectively. If only the first mode is considered, Eq. (4) becomes,

5 $$Y(x,t) = \phi (x)y(t)$$

The characteristic Eq. (6) is developed (see Appendix A) and solved to identify the eigenvalues $(\lambda )$ , which are the roots of the characteristic equation, to be able to estimate the natural frequencies. The characteristic equation may be written as,

6 $$\begin{gathered} 1 + \cos \,(\lambda )\,\cosh \,(\lambda ) - \lambda \,\frac{{M_{L} }}{{m_{s} L}}\,\left( {2\,\cos \,(\lambda )\,\sinh \,(\lambda ) + \sin \,(\lambda )\,\cosh \,(\lambda )} \right) \hfill \\ - \frac{{\lambda^{3} \,I_{L} }}{{m_{s} \,L^{3} }}\,\left( {\cosh \,(\lambda )\,\sin \,(\lambda ) + \sinh \,(\lambda )\,\cos \,(\lambda )} \right)\, + \frac{{\lambda^{4} \,M_{L} I_{L} }}{{m_{s}^{2} \,L^{4} }}\,\left( {1 - \cos \,(\lambda )\,\cosh \,(\lambda )} \right) = 0 \hfill \\ \end{gathered}$$

The characteristic Eq. (6) has unlimited number of roots $\lambda$, indicating that the system has an infinite number of eigenvalues, predicting an infinite number of natural frequencies. There is a unique mode of vibration associated with each eigenvalue. The vibration modes can be represented by the eigenfunction $\varphi \,(x)$.

For $r^{th}$ mode of vibration, eigenvalue is represented by $\lambda_{r}$ and the corresponding eigenfunction is given by $\varphi_{r} (x)$.

7 $$\varphi_{r} (x) = A_{r} \,\left[ {\cos \,\left( {\frac{{\lambda_{r} }}{L}\,x} \right) - \cosh \,\left( {\frac{{\lambda_{r} }}{L}\,x} \right) + \varsigma_{r} \left( {\sin \,\,\left( {\frac{{\lambda_{r} }}{L}\,x} \right) - \,\sinh \,\left( {\frac{{\lambda_{r} }}{L}\,x} \right)} \right)} \right]$$

where,

8 $$\varsigma_{r} = \frac{{\sin \,(\lambda_{r} ) - \sinh \,(\lambda_{r} ) + \lambda_{r} \,\frac{{M_{L} }}{{m_{s} \,L}}\left( {\cos \,(\lambda_{r} ) - \cosh \,(\lambda_{r} )} \right)\,}}{{\cos \,(\lambda_{r} ) + \cosh \,(\lambda_{r} ) - \lambda_{r} \,\frac{{M_{L} }}{{m_{s} \,L}}\left( {\sin \,(\lambda_{r} ) - \sinh \,(\lambda_{r} )} \right)\,}}$$

Applying the eigenfunction orthogonality principle, $A_{r}$ may be derived (see Appendix B) as,

9 $$A_{r} = \frac{1}{{\sqrt {\int_{0}^{{L_{b} }} {m_{s} \phi^{*2} (x)dx + \phi^{*2} (L_{b} )M_{L} + \phi^{*^{\prime}2} (L_{b} )I_{L} } } }},\,\,\,\,\,\phi_{r} (x) = A_{r} \phi^{*} (x)$$

The following formula yields the undamped natural frequency, also known as the eigenfrequency, of free oscillations for the $r^{th}$ vibration mode:

10 $$\omega_{r} = \lambda_{r}^{2} \,\sqrt {\frac{E\,I}{{m_{s} \,L^{4} }}}$$

A particular analytical method for modifying the turbine response to increase the effective power generation region is presented in this work. Based on aeroelasticity interaction numerical modeling phenomenon, the effective parameters on the turbine performance may be examined by a parametric analysis. Subsequently, the suggested tuning method attempts to keep the effective parameters at their best value while considering power generation. Two approaches are suggested for this purpose.

##### First approach: changing the effective length of the flexible stand (beam)

**Fig. 3:**
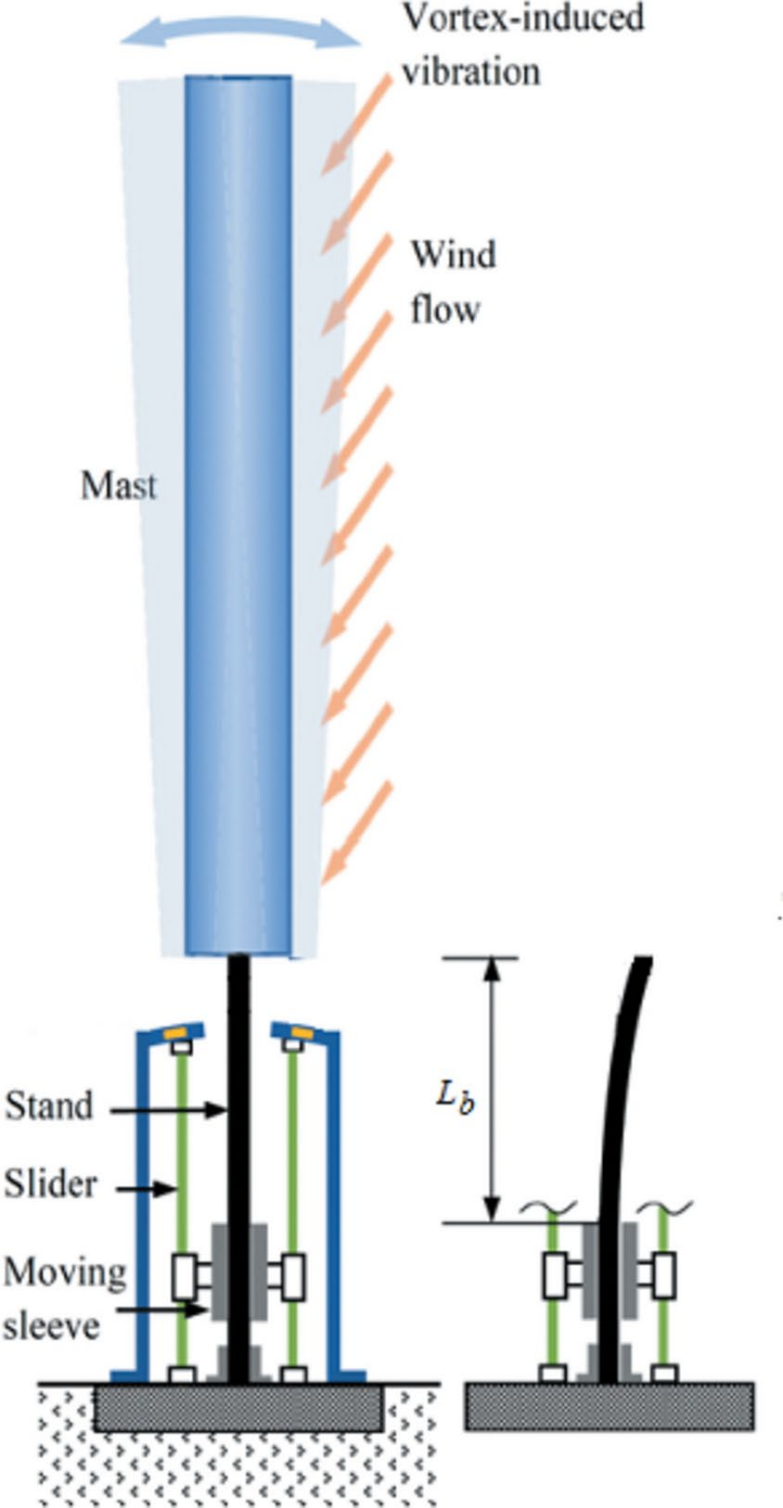
The vortex induced vibration bladeless wind turbine with adjusting sleeve of the flexible stand’s effective length.

As shown in Fig. 3, the vortex bladeless turbine consists of a rigid capture part, or cylindrical mast, on a flexible beam (stand) that is fixed to the floor. Significant energy transfer from the fluid to the structure occurs when operating within the lock-in region. The supporting sleeve can be moved up and down along two sliders using a linear actuator. In this study the mast is considered as a rigid body, while the beam is considered as an elastic component negligible inertia. A stable trend of around 0.2 is observed in the Strouhal number throughout a broad range of Reynolds numbers, $300 < R_{e} < 3 \times 10^{5}$^40^. This indicates that, within this range, the vortex shedding frequency changes linearly with the fluid flowing speed. When the structure’s fundamental frequency and the aerodynamic force’s frequency coincide, the oscillation’s amplitude increases significantly.

The proposed turbine design is modeled as a cantilevered uniform beam with a tip mass.

($M_{L} \,\,and\,\,I{}_{L}$). As a function of the stand effective length $(L_{b\, - \,eff} )$, the tip mass at lock-in region is a function of the stand’s effective length. The needed stand effective length may be determined using ${L_{b - eff}} = \lambda \sqrt[4]{EI/{m_s}\omega _s^2}$ by solving characteristic Eq. (6), which yields the eigenfrequency $\lambda$. To optimize the energy production rate of Vortex-Induced-Vibrations of the proposed bladeless wind turbine (VIV-BWT), it is essential to examine the effects of the turbine design parameters, geometric parameters of the stand and mast, on the lock-in phenomenon, considering the type of stand material.

##### Second approach: the use of the tuned mass mechanism

A tuned mass mechanism can be employed to achieve the second degree of structural adjustment. The proposed tuned mass mechanism consists of a linear actuator (represented by the guide rod and its base) that moves a tunable mass within the hollow mast, the characteristics of the proposed VIV-BWT, illustrated in Fig. 1a, are listed in Table 2.

This adjustable mass alters the natural frequency of the VIV-BWT energy harvester by changing the effective inertia of the cylindrical mast unit.

With a cantilever uniform beam configuration, the mass ($M_{L} \,\,and\,\,I_{L}$) at the end of the stand influences the solution of the characteristic Eq. (6). The tuned mass ($m_{t}$) and its location $s$ measured from the stand’s tip are two of the mast unit’s characteristics that affect $M_{L} \,\,and\,\,I_{L}$. To attain the lock-in condition, the natural frequency is adjusted to match the shedding frequency. For specified values of the stand cross-section and length, the value of eigenfrequency can be determined using Eq. (10). The location of the tuned mass, which is related to the stand length via the eigenfrequency value, is found by solving the characteristic Eq. (6) for the estimated $\lambda$. To meet the lock-in requirement, an iterative (trial and error) approach is used to determine the necessary effective length of the stand and corresponding tuned mass location, in order to achieve the minimum size of the turbine while maintaining smooth and accurate relationships between these variables and wind speed. Because there are several variables in the design of the bladeless wind turbine components that affect the lock-in condition, it is recommended to apply an optimal design technique.

#### Vortex-induced vibration (VIV) model

Two dynamic models form the dynamic vortex-induced vibration model; the first has been created for the wake oscillator and is linked to the second dynamic model of the structure oscillator.

##### Wake oscillator model

For an oscillating cylinder, because the mast is a rather long cylinder, the 2D-flow semi-empirical wake oscillator model is the most common. The oscillating lift coefficient $C_{L} (x,t)$ may be determined as follows^40^,

11 $$C_{L} \left( {x,t} \right) = Q\left( {x,t} \right) - \frac{2\,\alpha }{{D_{m} \,\omega_{s} }}\,\dot{Y}\left( {x,t} \right)$$

where $Q(x,t)$ is a dimensionless wake variable that describes the oscillating dynamics, $\dot{Y}(x,t)$ is the oscillation transverse velocity, $D_{m}$ is the diameter of the mast, $\omega_{s}$ is the vortex shedding angular frequency, and $\alpha$ is an empirical parameter. The second component to the right of Eq. (11) states that considerable structural movement has a negative effect on the amplitude of the oscillating lift coefficient. Triantafyllou et al.^42^ proposed involving this term based on findings from experiments. According to Hartlen and Currie^43^, the nonlinear van der Pol oscillator equation accurately describes the wake variable $Q(x,t)$, which is the excitation component of the changing lift coefficient. The nonlinear van der Pol oscillator equation is

12 $$\ddot{Q}\,\left( {x,t} \right) - \omega_{s} \,G\,\left( {C_{Lo}^{2} - 4\,Q^{2} (x,t)} \right)\,\dot{Q}\left( {x,t} \right) + \omega_{s}^{2} \,Q\,\left( {x,t} \right) = \frac{{\omega_{s} \,F}}{{D_{m} }}\,\dot{Y}\left( {x,t} \right)$$

G, and F are empirical parameters^40^. For stationary cylinders, $C_{Lo}$ is the amplitude of the fluctuating lift coefficient on a stationary cylinder.

##### Structure oscillator model (equation of motion)

The free-end displacement and rotation of the beam are expressed as $Y(L_{b} ,t)$ and $Y^{\prime}(L_{b} ,t)$, respectively. Assuming that the beam’s fundamental frequency is dominant, only the first mode may be studied in the present investigation. Assuming the parameter $\gamma$ is defined as the ratio of rotation to displacement at the beam end, the result is

13 $$\gamma = \frac{{\theta (L_{b} )}}{{y(L_{b} )}} = \frac{{Y^{\prime}(L_{b} ,t)}}{{Y(L_{b} ,t)}} = \frac{{\varphi^{\prime}_{1} }}{{\varphi_{1} }} = - \,\frac{{\lambda_{1} \,[\sin \,\lambda_{1} + \sinh \,\lambda_{1} - \varsigma_{1} \,(\cos \,\lambda_{1} + \cosh \,\lambda_{1} )]}}{{L_{b} \,[\cos \,\lambda_{1} - \cosh \,\lambda_{1} + \varsigma_{1} \,(\sin \,\lambda_{1} - \sinh \,\lambda_{1} )]}}$$

The superscript *(')* denotes the derivative with respect to. $x$. As can be seen, with a given tunable mass $(m_{t} )$, beam length $(L_{b} )$, and mass per unit length of the beam $(m_{s} )$, the parameter $\gamma$ may be determined by first calculating the eigenvalue $\lambda_{1}$ and then substituting it into Eq. (8) to calculate the modal parameter $\varsigma_{1}$. Equation (13) is then used to find the ratio $\gamma$ following $\lambda_{1}$ and $\varsigma_{1}$ are known.

There are two motion coordinates defined for the vibrating mast unit: displacement in the y-direction and mast angular movement. The two positions are linked together by the ratio $\gamma$, Eq. (13). As a result, we have one generalized coordinate that may be defined based on motion in the y-direction. In terms of kinetic energy (T), potential energy (U) and dissipative energy ($D_{e}$), the Lagrange equation may be represented as^50^,

14 $$\frac{d}{d\,t}\,\left( {\frac{\partial \,T}{{\partial \,\dot{Y}(x,t)}}} \right) - \frac{\partial \,T}{{\partial \,Y(x,t)}} + \frac{\partial \,U}{{\partial \,Y(x,t)}} + \frac{{\partial \,D_{e} }}{{\partial \,\dot{Y}(x,t)}} = \frac{\delta \,\,W}{{\delta \,Y(L_{b} ,t)}}$$

Substituting Eq. (5) into Eq. (14) yields

15 $$\frac{d}{d\,t}\,\left( {\frac{\partial \,T}{{\partial \,\dot{y}}}} \right) - \frac{\partial \,T}{{\partial \,y}} + \frac{\partial \,U}{{\partial \,y}} + \frac{{\partial \,D_{e} }}{{\partial \,\dot{y}}} = \frac{\delta \,\,W}{{\delta \,y}}$$

The kinetic, potential, and dissipative energies of the turbine may be estimated as follows:

16 $$\begin{gathered} T = \frac{1}{2}\,M_{b} \,\dot{y}^{2} (t)\,\, + \,\frac{1}{2}\,m_{m} \,\left[ {\left( {\varphi (L_{b} )\dot{y} + \overline{L}_{m} \,\varphi^{\prime}(L_{b} )\dot{y}\,\cos \,\theta } \right)^{2} + \left( {\overline{L}_{m} \,\varphi^{\prime}(L_{b} )\dot{y}\,\,\sin \,\theta } \right)^{2} } \right] \hfill \\ + \frac{1}{2}\,m_{t} \,\left[ {\left( {\varphi (L_{b} )\dot{y} + s\,\varphi^{\prime}(L_{b} )\dot{y}\,\cos \,\theta } \right)^{2} + \left( {s\,\varphi^{\prime}(L_{b} )\dot{y}\,\,\sin \,\theta } \right)^{2} } \right] \hfill \\ + \frac{1}{2}\,m_{r} \,\left[ {\left( {\varphi (L_{b} )\dot{y} + \overline{L}_{rod} \,\varphi^{\prime}(L_{b} )\dot{y}\,\cos \,\theta } \right)^{2} + \left( {\overline{L}_{rod} \,\varphi^{\prime}(L_{b} )\dot{y}\,\,\sin \,\theta } \right)^{2} } \right]\,\, \hfill \\ + \frac{1}{2}\,\varphi^{{\prime}{2}} (L_{b} )\,\left( {I_{m} + I_{rod} + I_{t} } \right)\,\dot{y}^{2} (t) \hfill \\ \end{gathered}$$

17 $$U = \frac{1}{2}\,K_{b} \,y^{2} - \,(m_{m} \,\overline{L}_{m} + m_{t} \,s + m_{r} \,\overline{L}_{rod} )\,g\,(\frac{{\varphi^{{\prime}{2}} (L_{b} )\,y^{2} }}{2})$$

18 $$D_{e} = \frac{1}{2}\,C_{b} \,\dot{y}^{2} (t)\,$$

where,

$$M_{b} = \int\limits_{0}^{{L_{b} }} {\varphi^{2} (x)\,m_{s} \,dx} \,\,,\,\,\,K_{b} = \int\limits_{0}^{{L_{b} }} {E\,I\,\varphi^{{\prime\prime}{2}} (x)\,\,dx} \,\,and\,\,C_{b} = \int\limits_{0}^{{L_{b} }} {C_{s} \,\,\varphi^{2} (x)\,\,dx}$$

In addition to the previously mentioned, the non-conservative work is the work of aerodynamic forces, represented by the y-direction lift force ($F_{L}$). The lift force caused by vortex shedding acting on the turbine’s body mast provided by

19 $$F_{L} = \int\limits_{{L_{b} }}^{{L_{b} + L_{s} }} {\frac{1}{2}\,\rho \,V^{2} \,D_{m} \,\,C_{L} (x,t)} \,dx$$

where the wake oscillator model at a time *t* determines the oscillating lift coefficient $C_{L} (x,t)$ at a cross-section $x$ away from its origin $O^{\prime}$. The non-conservative work of aerodynamic force may be given by,

20 $$\delta \,W = F_{L} \,\delta \,Y(L_{b} ,t) + M\,\delta \,\theta (L_{b} ,t)$$

where, $F_{L} \,\,and\,\,M$ are, respectively, the aerodynamic force and moment exerted from the mast on the tip of the beam to give the displacement $Y(L_{b} ,t)$ and rotation angle $\theta \,(L_{b} ,t)$.

Substituting Eq. (5) into Eq. (20) produces

21 $$\delta \,W = F_{L} \,\left( {\varphi \,(L_{b} )\,\,\delta \,y\,(t)} \right) + M\,(\varphi^{\prime}\,(L_{b} )\,\,\delta \,y\,(t))$$

And,

22 $$M = \int\limits_{{L_{b} }}^{{L_{b} + L_{m} }} {\frac{1}{2}\,\rho \,V^{2} \,D_{m} \,\,C_{L} (x,t)} \,(x - L_{b} )\,dx$$

The mode shapes of $Q(x,t)$ exactly match the mode shapes of $Y(x,t)$^40^;$Q(x,t) = \varphi (x)\,q(t)$. The velocity of the mast may be obtained as,

$$\begin{gathered} At\,\,\,\,L_{b} \le x \le \,(L_{b} + L_{m} ) \hfill \\ \dot{Y}(x,t) \cong \dot{Y}(L_{b} ,\,t) + (x - L_{b} )\,\dot{\theta }_{mast} \hfill \\ \end{gathered}$$

Then,

23 $$\left. \begin{gathered} \dot{Y}(x,t) = \left[ {\varphi (L_{b} ) + (x - L_{b} )\,\varphi^{\prime}(L_{b} )} \right]\,\dot{y}\,(t) \hfill \\ and\,\, \hfill \\ Q(x,t) = \left[ {\varphi (L_{b} ) + (x - L_{b} )\,\varphi^{\prime}(L_{b} )} \right]\,q\,(t) \hfill \\ \end{gathered} \right\}$$

The equation of motion can be obtained by substituting Eqs. (16)–(23) into Lagrange’s Eq. (15). For small angles, the trigonometric functions are approximated as $\sin \,\theta \approx \theta$ and $\cos \,\theta \approx 1$. By doing this, the equation of motion can be further simplified as follows:

24 $$M_{eq} \,\ddot{y}\,\left( t \right) + C_{eq} \,\dot{y}\,\left( t \right) + K_{eq} \,y\left( t \right) + CK_{nl} \,y\,\dot{y}^{2} = F_{eq} \,q\left( t \right)$$

where,

$$\begin{gathered} M_{eq} = \int\limits_{0}^{{L_{b} }} {\phi^{2} (x)m_{s} dx + (m_{m} + m_{t} + m_{rod} )\phi^{2} (L_{b} ) + \left( {I_{m} + I_{rod} + I_{t} + m_{m} \overline{L}_{m}^{2} + m_{t} s^{2} + m_{r} \overline{L}_{rod}^{2} } \right)} \phi^{^{\prime}2} (L_{b} ) \hfill \\ + 2\left( {m_{m} \overline{L}_{m} + m_{t} s + m_{r} \overline{L}_{rod} } \right)\phi (L_{b} )\phi{\prime} (L_{b} ) \hfill \\ \end{gathered}$$

$$\begin{gathered} C_{eq} = \int\limits_{0}^{{L_{b} }} {C_{s} \,\varphi^{2} (x)\,\,dx} + \frac{{\rho \,V^{2} \,\alpha }}{{\omega_{s} }}\left( {\int\limits_{{L_{b} }}^{{L_{b} + L_{m} }} {\left( {\varphi^{2} (L_{b} ) + \varphi (L_{b} )\,\varphi^{\prime}(L_{b} )\,(x - L_{b} )} \right)\,\,dx\,\,} } \right) \hfill \\ + \frac{{\rho \,V^{2} \,\alpha }}{{\omega_{s} }}\left( {\int\limits_{{L_{b} }}^{{L_{b} + L_{m} }} {\left( {\varphi \,(L_{b} )\,\varphi^{\prime}\,(L_{b} )\,(x - L_{b} )\, + \varphi^{{\prime}{2}} (L_{b} )\,(x - L_{b} )^{2} } \right)\,dx\,\,} } \right) \hfill \\ \end{gathered}$$

$$K_{eq} = E\,I\,\int\limits_{0}^{{L_{b} }} {\varphi^{{\prime\prime}{2}} (x)\,\,dx} - \,\left( {m_{m} \,\overline{L}_{m} + m_{t} \,s + m_{rod} \,\overline{L}_{rod} } \right)\,\varphi^{{\prime}{2}} (L_{b} )\,g$$

$$KC_{nl} = \, - \,\,\varphi (L_{b} )\,\varphi^{{\prime}{3}} (L_{b} )\,\left( {m_{m} \,\overline{L}_{m} + m_{t} \,s + m_{rod} \,\overline{L}_{rod} } \right)\,$$

$$\begin{gathered} F_{eq} = \frac{1}{2}\rho \,V^{2} D_{m} \,\int\limits_{{L_{b} }}^{{L_{b} + L_{m} }} {\left( {\varphi^{2} (L_{b} ) + \varphi (L_{b} )\,\varphi^{\prime}(L_{b} )\,(x - L_{b} )} \right)\,dx\,\,\,} \hfill \\ + \frac{1}{2}\rho \,V^{2} D_{m} \,\,\int\limits_{{L_{b} }}^{{L_{b} + L_{m} }} {\left( {\varphi (L_{b} )\,\varphi^{\prime}(L_{b} )\,(x - L_{b} ) + \varphi^{{\prime}{2}} (L_{b} )\,(x - L_{b} )^{2} } \right)\,dx\,} \hfill \\ \end{gathered}$$

Substituting Eq. (23) into Eq. (12), the van der Pol oscillator equation can be expressed as,

25 $$\begin{gathered} \left[ {\varphi \,(L_{b} ) + (x - L_{b} )\,\varphi^{\prime}(L_{b} )} \right]\,\ddot{q}\,(t) - \omega_{s} \,G\,C_{Lo}^{2} \,\left[ {\varphi \,(L_{b} ) + (x - L_{b} )\,\varphi^{\prime}(L_{b} )} \right]\,\dot{q}\,(t) \hfill \\ + 4\,\omega_{s} G\,\left[ {\varphi \,(L_{b} ) + (x - L_{b} )\,\varphi^{\prime}(L_{b} )} \right]^{3} \,q^{2} (t)\,\dot{q}(t) + \omega_{s}^{2} \,\left[ {\varphi \,(L_{b} ) + (x - L_{b} )\,\varphi^{\prime}(L_{b} )} \right]\,q(t) \hfill \\ = \omega_{s} \,F\,\left[ {\varphi \,(L_{b} ) + (x - L_{b} )\,\varphi^{\prime}(L_{b} )} \right]\,\frac{{\dot{y}\,(t)}}{{D_{m} }}\hfill \\ \end{gathered}$$

Let,

26 $$h\,(x) = \varphi \,(L_{b} ) + (x - L_{b} )\,\varphi^{\prime}(L_{b} )$$

Substituting (26) into (25) and integrate from $L_{b} \,\,to\,\,(L_{b} + L_{m} )$, the reduced model of the nonlinear van der Pol equation is

27 $$\,\ddot{q}\,\left( t \right) + B_{1} \,\dot{q}\,\left( t \right) + B_{2} \,q\,\left( t \right) + B_{3} \,\,q^{2} \left( t \right)\,\dot{q}\,\left( t \right) = B_{4} \,\dot{y}\,\left( t \right)$$

where,

$$\begin{gathered} B_{1} = - \,\omega_{s} G\,C_{Lo}^{2} \hfill \\ B_{2} = \omega_{s}^{2} \hfill \\ \end{gathered}$$

$$B_{3} = 4\,\omega_{s} \,G\,\,\left( {\frac{{\int\limits_{{L_{b} }}^{{L_{b} + L_{m} }} {h^{3} (x)\,dx} }}{{\int\limits_{{L_{b} }}^{{L_{b} + L_{m} }} {h\,(x)\,dx} }}} \right)$$

$$B_{4} = \omega_{s} \,\left( {\frac{F}{{D_{m} }}} \right)$$

##### Power transmitted to the BWT (P_m_)

The available power per unit length of the mast is expressed as,

$$dP = d\,F_{a} (x,t)\,\dot{Y}(x,t)\,\, = \frac{1}{2}\,\rho \,V^{2} D_{m} \,C_{L} (x,t)\,dx\,\,\dot{Y}(x,t)$$

$$\,C_{L} (x,t) = Q\,(x,t) - \frac{2\,\alpha }{{D_{m} \,\omega_{s} }}\,\dot{Y}\,(x,t)$$

Integration throughout the mast length $L_{m}$ of the turbine should be performed to calculate the resulting mechanical power transferred to the turbine $P_{m}$. The power $P_{m}$ may be written in a simple form as,

28 $$P = B_{6} \,q(t)\,\dot{y}\,(t) - B_{7} \,\dot{y}^{2} (t)$$

With

$$B_{6} = \frac{1}{2}\,\rho \,V^{2} D_{m} \,\int\limits_{{L_{b} }}^{{L_{b} + L_{m} }} {\left[ {\varphi (L_{b} ) + (x - L_{b} )\,\varphi^{\prime}(L_{b} )} \right]^{2} \,dx}$$

$$B_{7} = \,\,\frac{{\rho \,V^{2} \,\alpha }}{{\omega_{s} }}\,\int\limits_{{L_{b} }}^{{L_{b} + L_{m} }} {\left[ {\varphi (L_{b} ) + (x - L_{b} )\,\varphi^{\prime}(L_{b} )} \right]^{2} \,dx}$$

##### Mechanical efficiency of the BWT ($\eta_{m}$)

The mechanical efficiency, defined as,$\eta_{m} = \frac{{P_{m} }}{{P_{f} }}$ is the ratio of mechanical power to available fluid power. $P_{f}$ is the available fluid power, $P_{f} = 0.5\,\rho \,A_{s} V^{2}$, which estimated based on the swept area $A_{s}$ using^44^,

29 $$A_{s} = \left( {2\,L_{m} \,\left( {\frac{{D_{m} }}{2} + Y(L_{b} ,\,t)} \right) + L_{m}^{2} \,\tan \,(\theta_{\max } )} \right)$$

##### The empirical parameters $C_{Lo} \,,\,\,G\,,\,\,and\,\,F$

Skop and Balasubramanian^40^ demonstrated that the model’s empirical parameters are linked to the physical mass and damping parameters which define structural properties. The value $C_{Lo}$ for a circular section has been taken as $C_{Lo} = 0.28$, as given by Protos et al.^45^. The value of $S_{t}$ is chosen to be $S_{t} = 0.21$, which correlates to the subcritical region of Reynolds number, approximately from $3 \times 10^{2}$ to $3 \times 10^{5}$. The stall parameter $\alpha$ is set between 0.17 and 0.19 to provide some degree of agreement with the experimentally obtained findings. Thus, 0.183 has been chosen as the value of $\alpha$. For simulating vortex shedding from a mechanically oscillated cylinder, these value for $\alpha$ yields a value of 0.2 for $G\,C_{Lo}^{2}$^40^. The van der Pol equation was used together with this value of $G\,C_{Lo}^{2}$ by Noack et al.^46^ and Balasubramanian and Skop^47^ to describe the vortex shedding out of cylinders in a non-uniform flow field.

## Numerical simulation and model validation

**Fig. 4:**
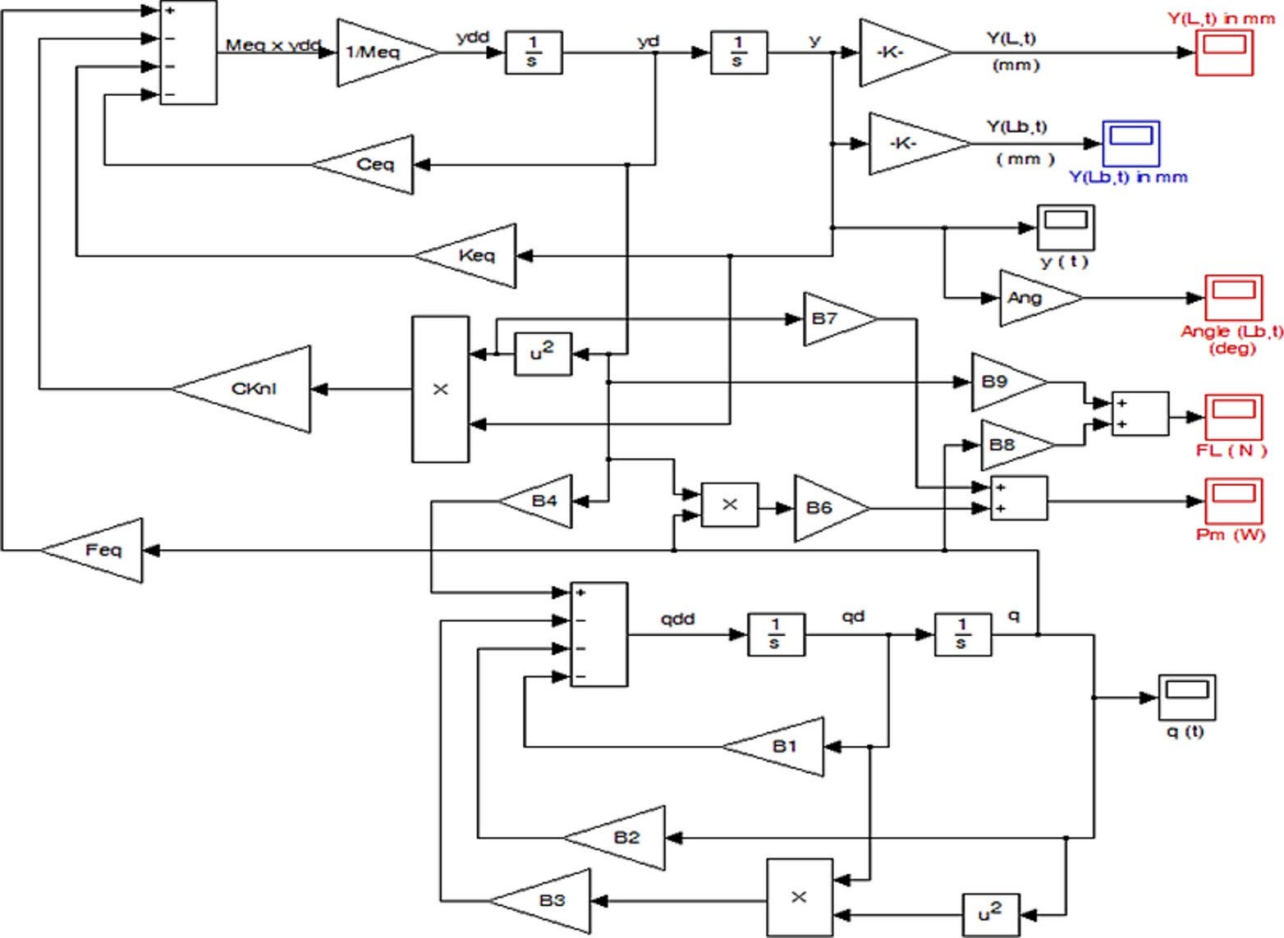
Simulink model.

Simulation of Dynamic Systems using MATLAB R2020a and Simulink R2020a is an in-depth examination of continuous simulation, providing all the necessary information and requirements in a single source. BWT dynamics is the study of a turbine’s response to wind. Understanding and managing turbine dynamics is essential for performance enhancement because they provide a wide range of features and capabilities for constructing, evaluating, and verifying complex systems. MATLAB and Simulink serve as valuable tools for modeling and simulating BWT dynamics. Figure 4 shows the Simulink model utilized to solve the turbine’s nonlinear dynamic equations, as outlined in Eqs. (24) and (27). Through this Simulink model, we obtained the turbine’s dynamic response, with key outputs including the lifting force, the amplitude of vibration at the mast’s free end, and the power supplied to the turbine.

To run the Simulink model, two MATLAB M. files must be created and prepared. The first program is written to use the MATLAB symbolic toolbox to obtain mathematical expressions for the various constants and derived parameters in the dynamic model’s governing equations ($M_{b} \,\,,\,\,K_{b} \,,\,\,\,C_{b} \,\,,\,\,F_{L} \,\,,\,\,M\,\,,\,\,M_{eq} \,,\,\,K_{eq} \,,\,\,C_{eq} \,,\,\,KC_{nl} \,,\,\,F_{eq}$ and $B_{1} \,,\,\,B_{3} \,,\,\,B_{4} \,,\,\,B_{6} \,,\,\,B_{7} \,$). The derived mathematical formulas are employed in the second MATLAB M-file, which contains all the turbine’s parameters such as mast diameter, mast thickness, mast length, stand length, stand diameter, stand thickness, flexural modulus of the stand material, and the empirical values G, $C_{Lo}$ and G. The second program must be executed before using the Simulink model illustrated in Fig. 4.

### Model validation

**Fig. 5:**
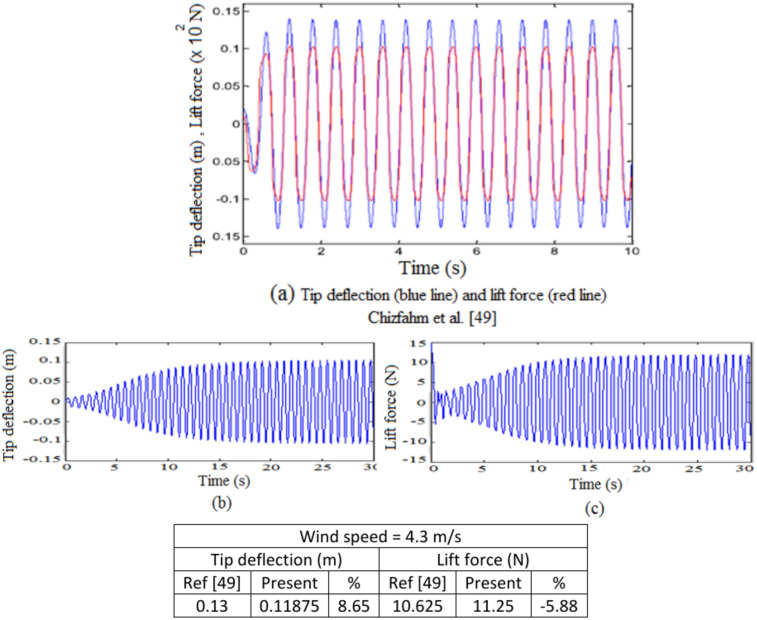
Time-based response comparison.

The current model was validated by using previously published research papers. The key aspects of the current methodology to be compared with previous studies are the turbine natural frequency, the time response of the entire lift force and mast tip displacement. The first approach validation shows high accuracy with Chizfahm et al.^49^. Who investigated the dynamic behavior of four configurations of vortex-induced vibrations in a bladeless wind turbine (BWTs), one of which mirrored to the current BWT. They presented the structure as a clamped-free Euler–Bernoulli beam to analyze lock-in phenomena, substituting the mast’s mass and inertia, as well as the distributed fluctuating lift force, with a resultant point force and a resultant point moment on the stand’s tip. They reported that the lock-in phenomena occur at wind speed of $V = 4.3\,m/s$ and stand length of *0.15 m*. Our model, employing identical turbine dimensions and characteristics, the first approach in the current model yielded a stand effective length of *0.1475* m at resonance and the same wind speed, demonstrating a *-1.67%* difference in required stand length, suggesting that the lock-in occurrence around the resonance. Consequently, validation of the first approach of this current study for predicting the natural frequency and the required stand’s effective length against results from^49^ revealed close correspondence, affirming the high accuracy of our model. At a wind speed of $V = 4.3\,m/s$, Fig. 5 illustrates the steady time-based responses of lift force and mast tip displacement. Figure 5a shows the outcomes obtained in^49^, Fig. 5b,c, shows the results of the current dynamic model of our study.

**Fig. 6:**
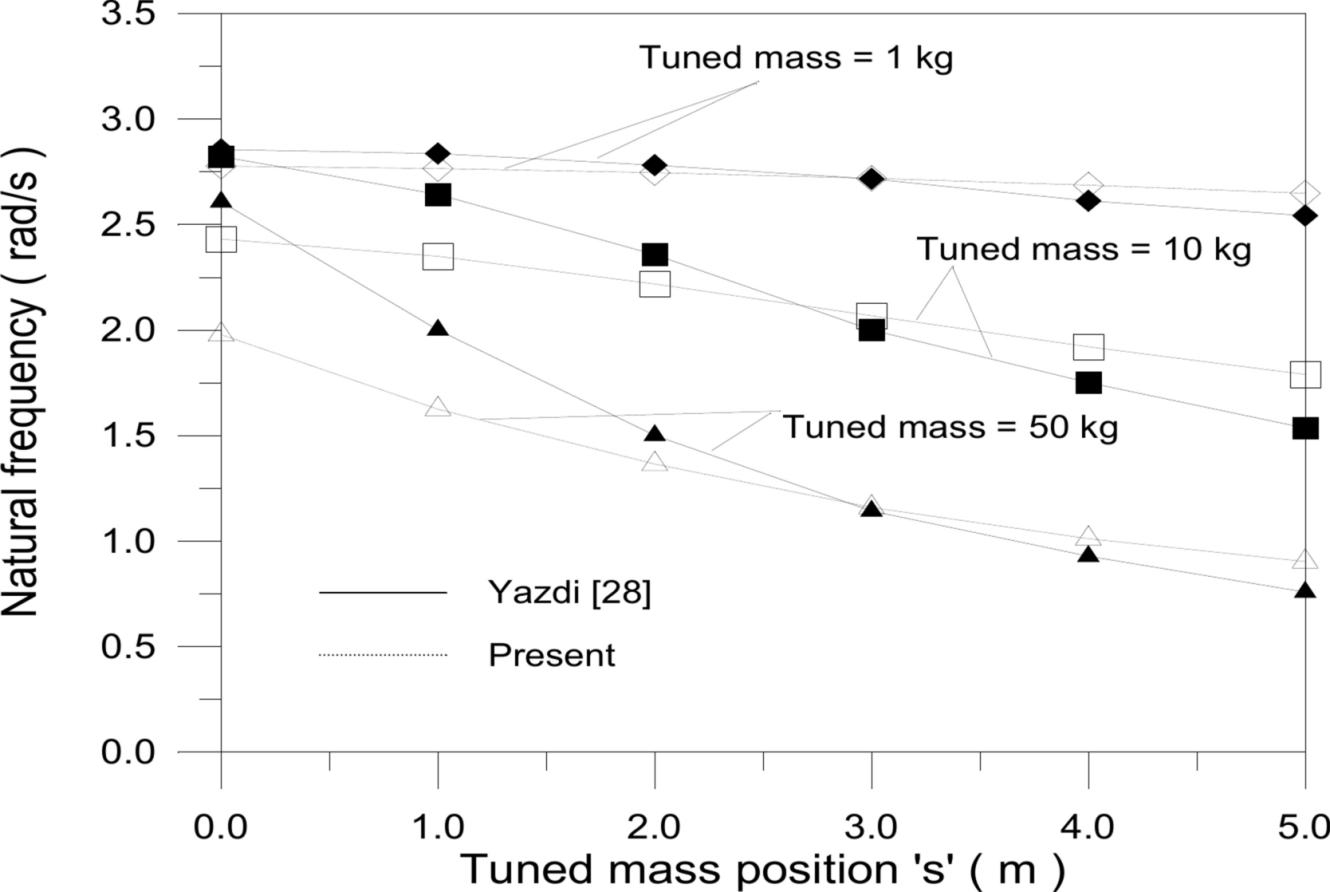
Tuned mass position comparison.

The second validation process involved an examination of the study conducted by Yazdi^32^, which focused on investigating the impact of a tuned mass on the natural frequency of the bladeless wind turbine (BWT). which moves along the axis of the hollow cylindrical mast, with the tuned mass location (s) measured from the stand’s tip. The mast had a mass of 10 kg, and tuned mass values of 1, 10, and 50 kg were examined. However, employing excessively large tuned mass values may not be practically feasible due to the considerable load they impose on the turbine’s supporting elements. Yazdi^32^ modeled the stand with a square cross-section as a multilayered clamp-free beam partially covered by piezoelectric patches on both sides. His model accounted for the impact of these patches on the stand’s potential and kinetic energy. By comparing the change in natural frequency for various tuned masses using^32^ model and the current study model, close agreement was observed, as depicted in Fig. 6. Table 3 summarizes the findings and highlights the largest percentage difference alongside the corresponding tuned mass location. Discrepancies in results can be attributed to the inclusion of piezoelectric patches in^32^, which was not considered in the current study. Both Yazdi’s model^32^ and this study demonstrate that the tuned mass location significantly influences the natural frequency, especially as the tuned mass value increases. Despite minor deviations, particularly at low tuned mass values, the overall agreement between the two models is satisfactory.

**Table 3:** Comparison of the natural frequency versus the location of the tuned mass.

$m_{t} \,(kg)$	$s\,\,(m)$	Yazdi^28^	Present	% difference
1	0	2.854	2.776	− 2.75
	5	2.541	2.647	4.1
10	0	2.821	2.43	− 13.86
	5	1.535	1.788	16.52
50	0	2.607	1.978	− 24.127
	5	0.758	0.902	19

## Results and discussion

### First approach: changing the effective length of the flexible beam

Optimizing the energy production rate of Vortex-Induced-Vibrations of the proposed bladeless wind turbine (VIV-BWT) by applying the first approach (elastic tuning mechanism) requires a detailed analysis of how turbine design parameters, geometric parameters of the stand and mast, impact the lock-in phenomenon, particularly in relation to the chosen stand materials mechanical properties (flexural modulus).

#### Effect of geometric parameters on stand effective length

**Fig. 7:**
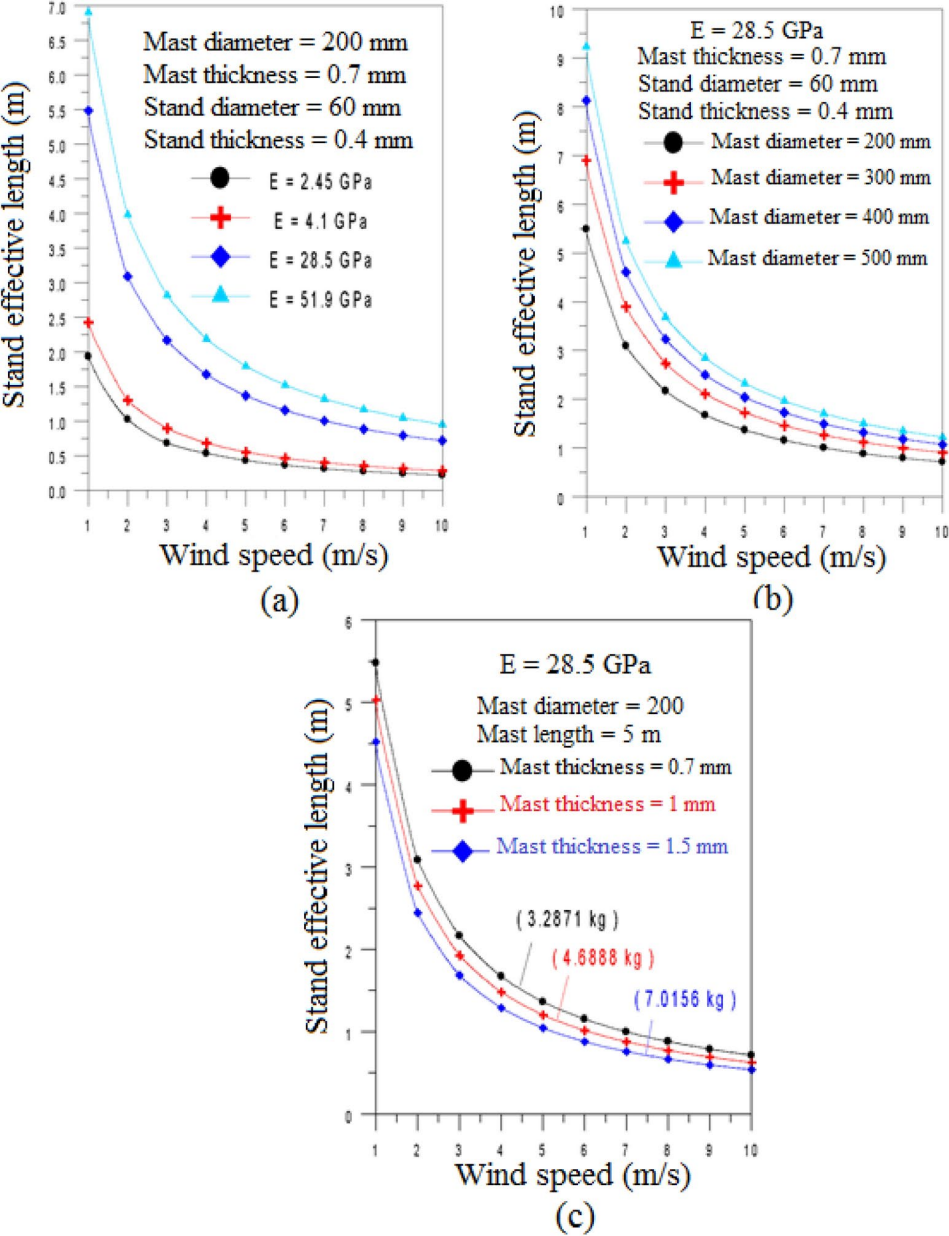
The variation of the effective length of the stand $L_{b\, - \,eff}$ versus the wind speed for (**a**) different flexural modulus of materials; (**b**) different mast diameters; (**c**) different mast masses.

By adjusting the elastic member length, the application of elasticity tuning can broaden the effective wind speed range while maintaining the lock-in state across varying wind conditions. For the specified turbine dimensions 200 mm in diameter, 0.7 mm in thickness, and 5 m in length, with a flexural modulus of E = 51.9 GPa. Figure 7a shows that the effective stand length decreases as wind speed increases, depending on the stand’s flexural modulus. As the flexural modulus increases, the required effective stand length also increases. At lower wind speeds, up to 3 m/s, the necessary stand length varies significantly, especially with high flexural modulus composite materials. The study finds that for high flexural modulus of E = 51.9 GPa, the stand length varies from 6.9072 m at 1 m/s to 0.9469 m at 10 m/s. For a lower flexural modulus of E = 2.45 GPa, the effective stand length ranges from 1.9297 m at 1 m/s to 0.2185 m at 10 m/s. The choice of stand material is critical, as it depends on the required maximum flexural strength.

The mast diameter plays a crucial role in the design of VIV-BWTs. As shown in Fig. 7b, the variation of effective stand length with wind speed for different mast diameters, ranging from 200 to 500 mm, for E = 28.5 GPa. Results shows an increase in mast diameter requires a corresponding increase in the effective stand length. This relationship indicates that as wind speed decreases, the required stand length becomes longer. This trend is consistent across all tested mast diameters, with larger diameters leading to a more substantial increase in the minimum effective stand length, while smaller diameters result in a reduced minimum length requirement.

Mast thickness, which affects the mast’s mass, is the final variable analyzed. Figure 7c results were obtained for mast thickness values ranging from 0.7 mm to 1.5 mm. Findings demonstrate that increasing the mast thickness results in a slight reduction in the effective stand length needed at various wind speeds. Figure 7a–c demonstrate that the lock-in phenomenon can be achieved by adjusting the length of the elastic element (stand). However, the effective length required is substantial, especially at low wind speeds.

#### Dynamic response for a bladeless wind turbine employing the elastic tuning mechanism

**Fig. 8:**
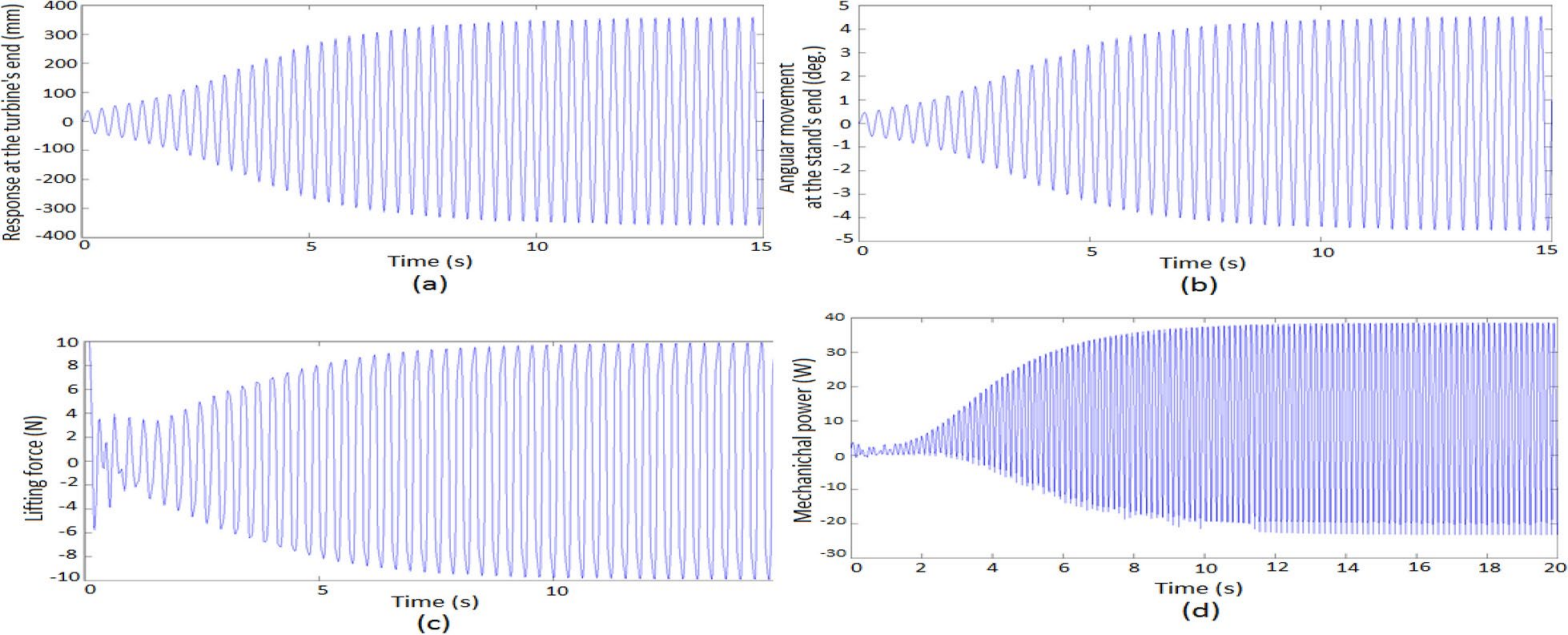
Dynamic response of the VIV-BWT, (Elastic tuning mechanism). (**a**) displacement; (**b**) angular movement; (**c**) lifting force, mechanical power ($E = 28.5\,\,GPa\,\,,\,\,V = 6\,m/s\,\,,\,\,L_{b} = 1.1526\,m\,\,,\,\,\omega_{n} = \omega_{s} = 39.5829\,\,rad/s$).

The dynamic response of the vortex-induced vibration bladeless wind turbine (BWT) energy harvester, incorporating an elastic tuning mechanism to adjust the stand’s effective length, was analyzed. The examined BWT has the following dimensions and material properties: mast diameters of 0.3 m and 0.2 m, mast thickness of 0.7 mm, mast length of 5 m, stand flexural modulus of 28.5 GPa, stand diameter of 60 mm, and stand thickness of 0.4 mm. The stand effective length required to achieve the lock-in condition, based on the estimated lock-in velocity, is summarized in Table 4. Figure 8 illustrates the dynamic response of the turbine using the geometric parameters outlined in Table 2 for the mast and stand flexural modulus of $E = 28.5\,GPa\,\,.$

**Table 4:** Stand effective length related to the lock-in wind speed.

$V_{Lock - in}$ (m/s)	$L_{b}$(m)	
	$D_{m} = 0.2$	$D_{m} = 0.3$
1	5.4829	6.897
2	3.0873	3.8954
3	2.1642	2.7316
4	1.6713	2.11
5	1.3635	1.7219
6	1.1526	1.4559
7	0.9987	1.2619
8	0.8814	1.114
9	0.7889	0.9974
10	0.7142	0.9031

Figure 8a,b display the transverse displacement of the VIV-BWT tip, $Y(L_{b} + L_{m} \,,\,\,t)$, and the angular movement at the stand tip $Y^{\prime}(L_{b} \,,\,t)$ at a wind speed of $V_{Lock - in} = 6\,m/s$. The amplitude of the turbine tip and the maximum angular movement in RMS is 0.318 m and 4.6° in the steady state.

Figure 8c displays the time response of the lift force, indicating self-sustaining, steady, and nearly harmonic oscillation, which are fundamental properties of vortex-induced vibration^48^. Figure 8d illustrates the turbine’s mechanical power output, with a steady-state RMS power output of $P_{m} = 36.12\,\,W$.

#### Effect of elastic tuning mechanism on bladeless wind turbine performance

**Fig. 9:**
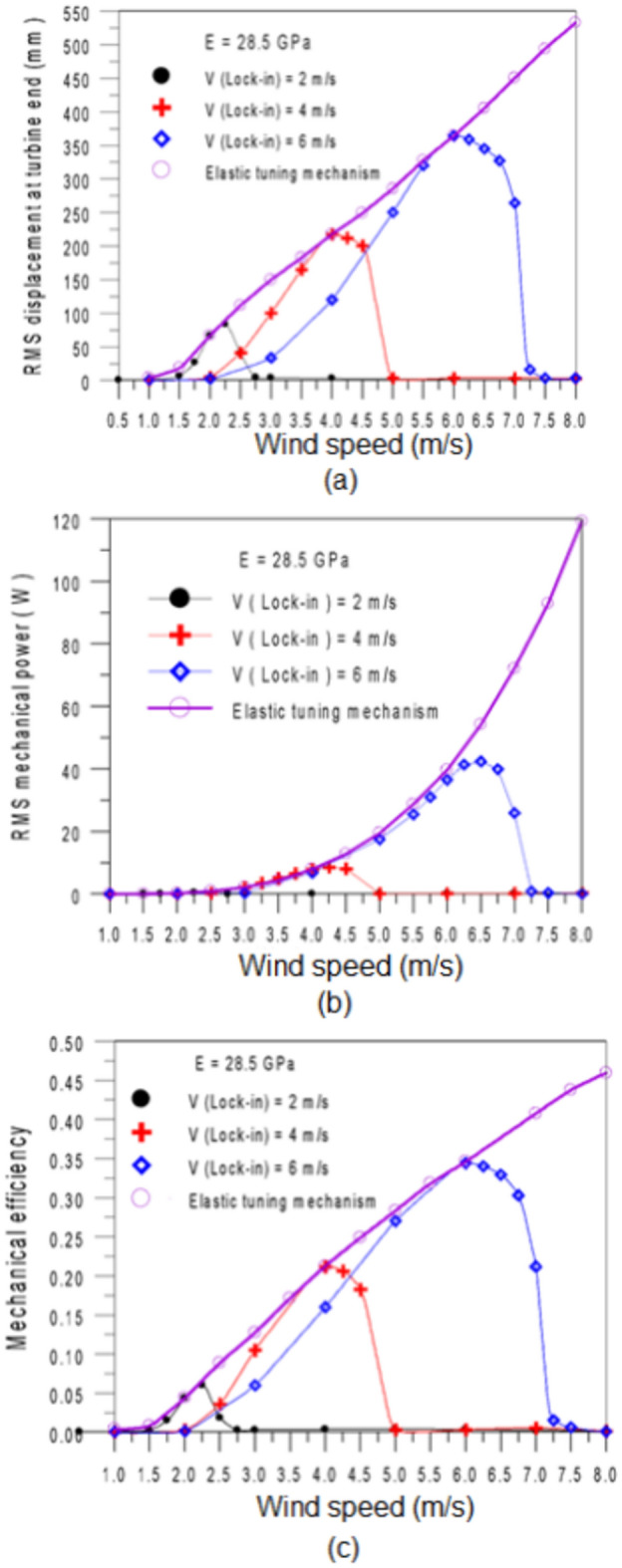
Effect of the elastic tuning mechanism on the performance of the turbine. (**a**) RMS displacement of mast end, (**b**) RMS mechanical power, (**c**) mechanical efficiency.

Figure 9 presents a comparison of the dynamic response, output mechanical power, and mechanical efficiency of the BWT, both with and without applying the elastic tuning mechanism.

For a traditional bladeless wind turbine, the stand is fixed at a specified lock-in wind speed (2 m/s, 4 m/s or 6 m/s) and remains unchanged with wind speed variation ranging from 1 to 8 m/s. in this configuration, the adjustable turbine operates similarly to a conventional one. However, the results show that outside the lock-in zone, the collected power rapidly decreases due to a loss of synchronization between the vortex-shedding frequency and the natural frequency (Fig. 9b). This behavior is also observed in the vibration amplitude at the mast end (Fig. 9a) and in the mechanical efficiency (Fig. 9c).

In contrast, when the elastic tuning mechanism is employed, allowing the sleeve to move up and down, the turbine’s performance significantly improves across the wind speed range. At a wind speed of 8 m/s, the output mechanical power markedly increases to $P_{m} (RMS) = 119.26\,W$. The same findings are observed for both the mast end vibration amplitude. The mechanical efficiency shows a 99.2% increase at 7 m/s compared to conventional BWT. Findings illustrates how effectively the elastic tuning mechanism enhances overall performance, particularly across varying wind speeds.

The elastic tuning mechanism findings demonstrate that while the lock-in phenomenon can be achieved by adjusting the elastic element’s length (stand) and consequently enhance all BWT performance, applying this mechanism requires a substantial effective length, particularly at low wind speeds, this approach is only practical when the stand length remains significantly shorter than the mast. Therefore, relying solely on the elastic stand adjustment is not recommended; an additional structural adjustment is necessary. Second approach (the tuned mass mechanism) can be employed to achieve the second degree of structural adjustment. Consequently, resonance can be enhanced by adjusting the turbine’s natural frequency closer to the vortex shedding frequency. There was qualitative agreement in the turbine’s performance with varying wind speeds when comparing the results of Magdy et al.^33^ with the performance of the proposed turbine if the effective length of the beam was changed. This was true even though the operating conditions, the mathematical model used, and the inclusion of the electric generator in the mathematical model in^33^ differed.

### Second approach: employing the tuned mass mechanism

The second approach involves the innovative use of a tuned mass mechanism to precisely adjust the resonance conditions of the bladeless wind turbine. Changing the tunable mass position by moving it within the hollow mast as shown in Fig. 1, This adjustable mass alters the natural frequency of the VIV-BWT energy harvester by changing the effective inertia of the cylindrical mast unit, consequently achieving resonance.

**Fig. 10:**
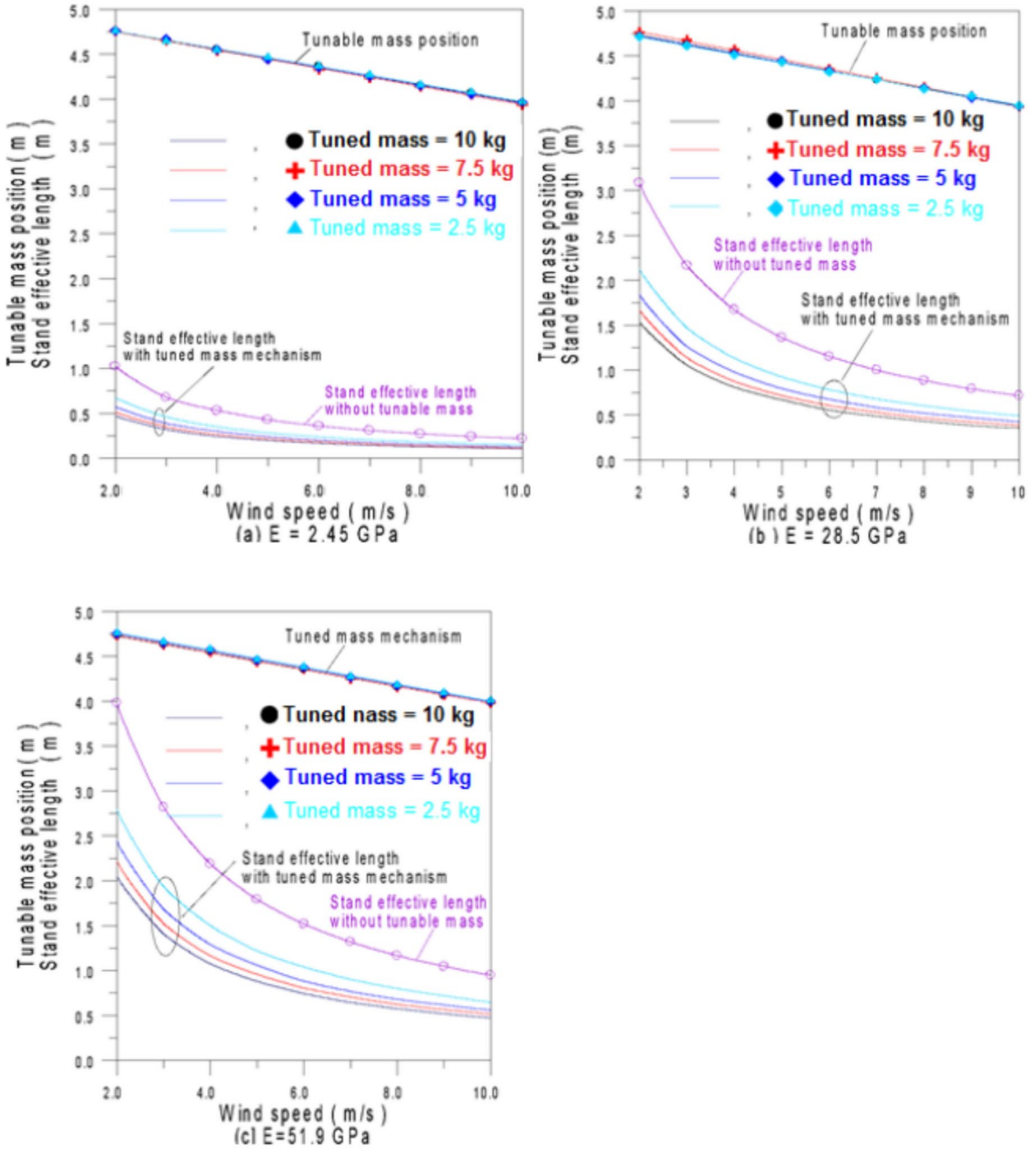
Stand effective length and adjustable mass location for varied Flexural modulus values and wind speeds.

Figures 10a–c shows the relationship between the tuned mass position and the stand’s effective length for a mast diameter of 0.2 m and varying flexural moduli. This relationship enables the achieving of the lock-in condition across a range of wind speeds from 2 to 10 m/s, depending on the value of the tuned mass.

Findings demonstrate that applying the tuned mass mechanism reduces the effective stand length by presenting the stand effective lengths both with and without applying the tuned mass. Using these results, BWTs can be optimized to achieve resonance at specific wind speeds by positioning the mass at precise locations aligns with its stand length.

**Fig. 11:**
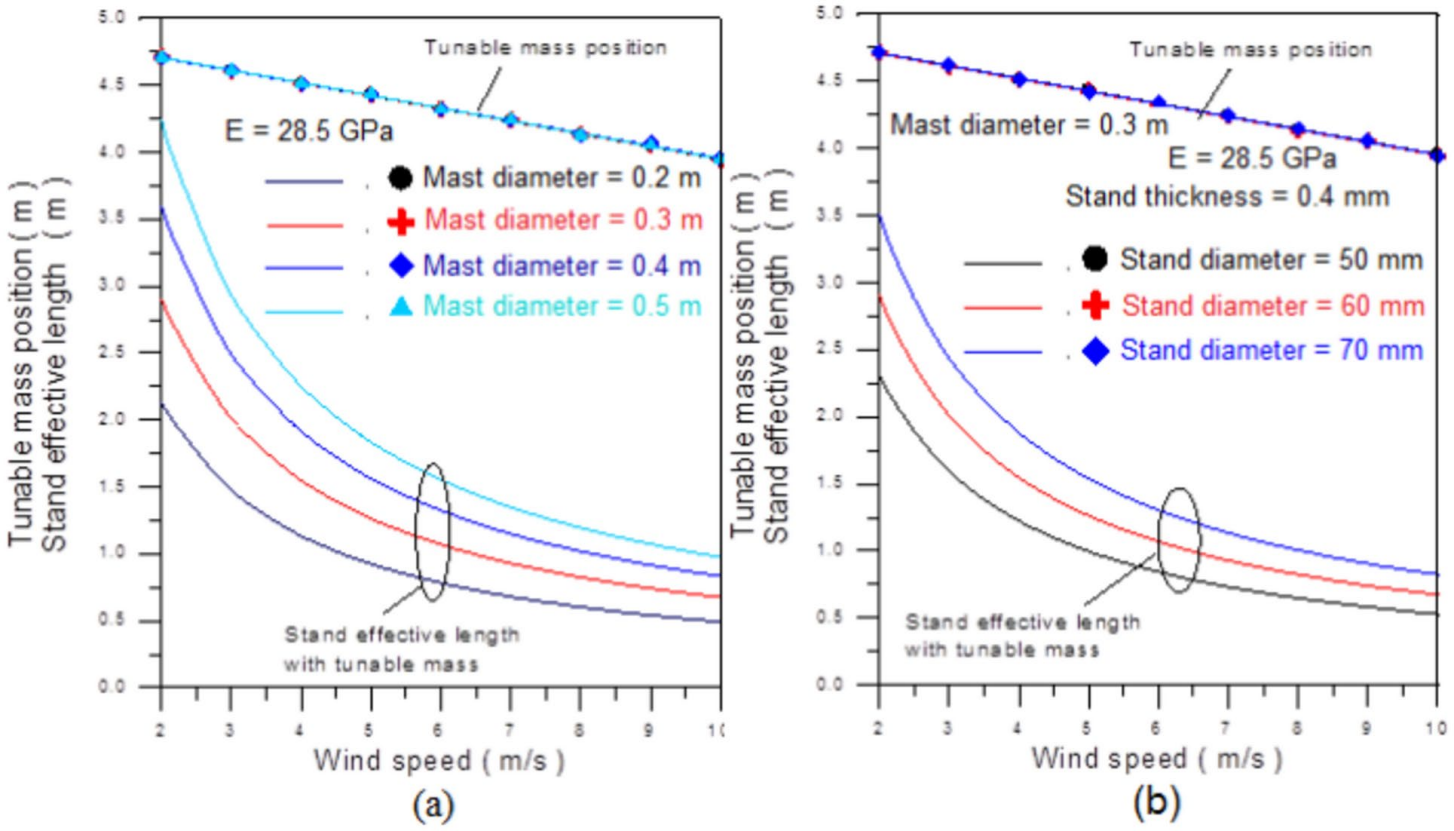
Stand effective length and adjustable mass position versus wind speed with varying; (**a**) mast diameter and (**b**) varying stand diameter.

Figure 11 shows that the tuned mass mechanism can be employed for different mast and stand diameters using similar tuning patterns. Figure 11a illustrates that as the mast diameter decreases, the required effective length decreases. Similarly, Fig. 11b demonstrates that a reduction in stand diameter corresponds to a decrease in required effective length.

**Fig. 12:**
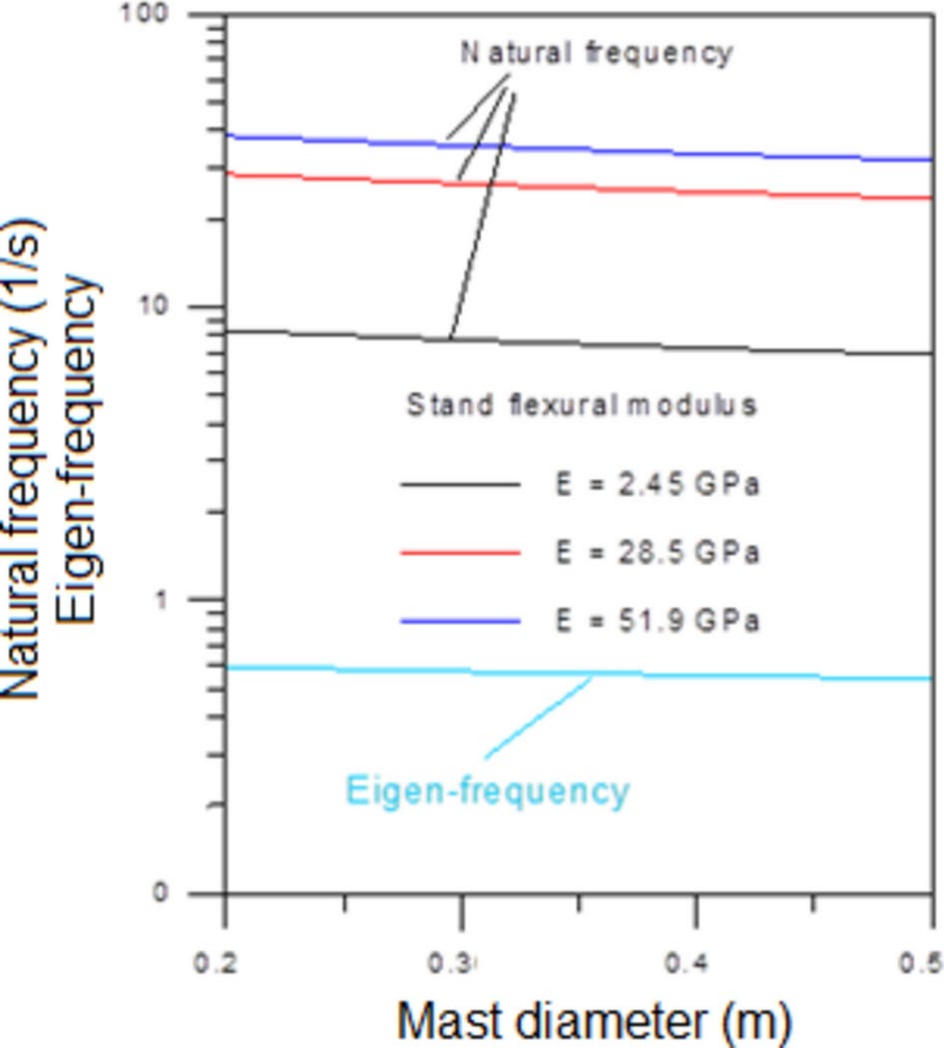
Eigen-frequency and natural frequency using various values of stand flexural modulus.

Figure 12 illustrates the variation of the Eigen-frequency with mast diameter (depends on $M_{L} \,\,and\,\,I_{L}$) while the natural frequency is influenced by stand cross-section, stand length and stand flexural modulus as indicated by Eq. (10). In general, it has been found that using a stand material with a relatively high flexural modulus, the tuned mass mechanism significantly reduces the required length of the stand, especially for relatively low wind speeds. As the tuned mass increases, the required effective length of the stand decreases. The effective length of the stand varies with wind speed via an exponential decay relationship, while the position of the tuned mass varies with wind speed in a linear manner. According to the proposed model to regulate both the tuned mass mechanism and the elastic tuning mechanism, the turbine can meet the lock-in requirement across a wide range of wind speeds.

### Integration of elastic tuning and tuned mass mechanism

**Fig. 13:**
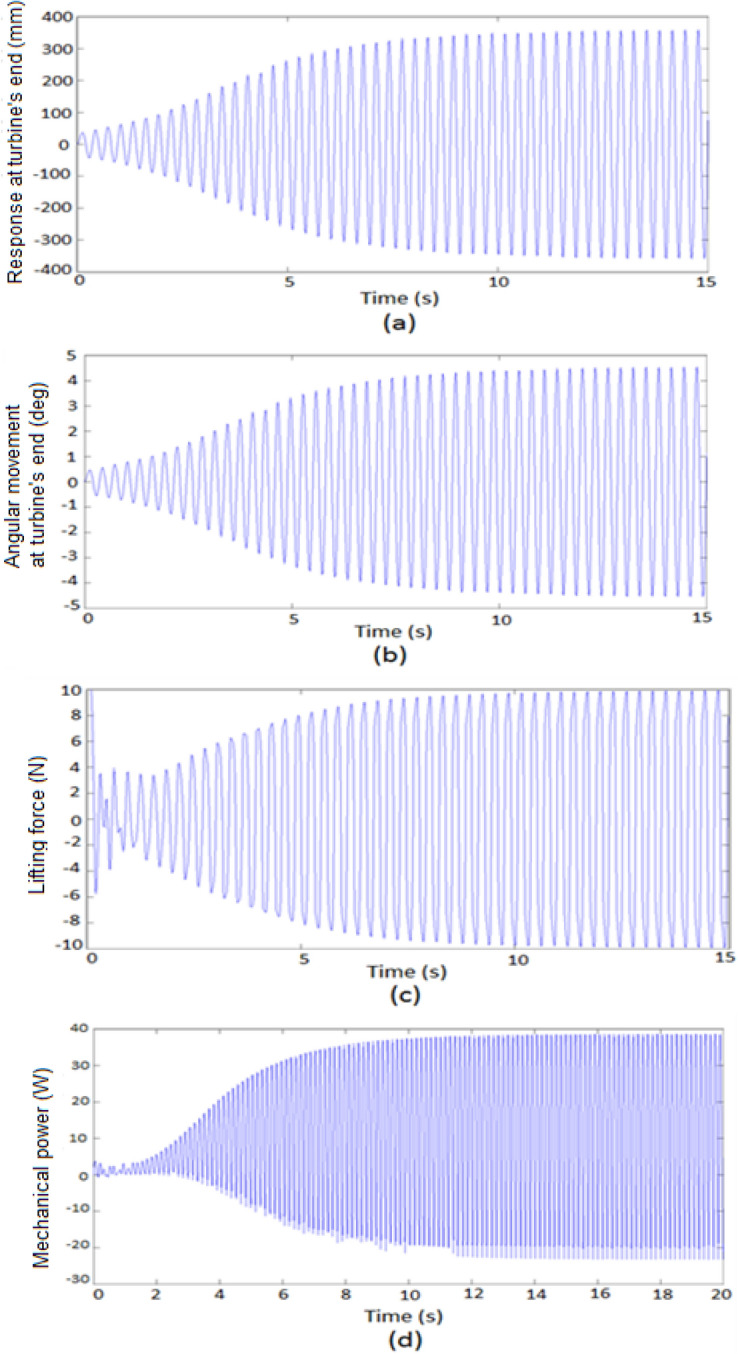
Dynamic response of the VIV-BWT using elastic tuning mechanism and mass tuning mechanism with tuning mass of 2.5 kg at wind speed of 6 m/s. (**a**) displacement; (**b**) angular movement; (**c**) lifting force, (**d**) mechanical power. $$\,\left( \begin{gathered} L_{b} = 0.782\,m\,,\,\,m_{t} = 2.5\,kg\,\,,\,\,s = 4.32\,m\,\,,\, \hfill \\ \,\omega_{n} = 19.5586\,\,rad/s\,\,,\,\,\omega_{s} = 39.5841\,rad/s \hfill \\ \end{gathered} \right)$$.

To investigate the combined effects of the elastic tuning and tuned mass mechanisms for $m_{t} = 2.5\,kg$, the performance of the turbine was analyzed at a wind speed of 6 m/s. Figure 13 illustrates the turbine’s performance under these conditions, showing an effective stand length of 0.872 m and a tuned mass position at 4.23 m, achieving resonance with the vortex shedding frequency. To evaluate the effects of using the tuned mass mechanism, define the tuned mass ratio as the ratio of the mass of the tuned mass mechanism (represented by the tuned mass $m_{t}$ and the guide rod mass $m_{r}$) to the total mass of the cylindrical mast unit, given by $M_{r} = {{(m_{t} + m_{r} )} \mathord{\left/ {\vphantom {{(m_{t} + m_{r} )} {(m_{m} + m_{t} + m_{r} )}}} \right. \kern-0pt} {(m_{m} + m_{t} + m_{r} )}}$. The time frame response of the transverse displacement at the free end of the mast, the angular movement at the tip of the stand, the integrated lift force and the integrated mechanical power are shown in Fig. 13a–d.

Using the tuned mass mechanism significantly enhances the turbine’s dynamic performance by increasing both the response amplitude and the output mechanical power. This improvement is evident when comparing the results in Fig. 13 which includes the tuned mass mechanism to those results obtained in Fig. 8 which only employs the elastic tuning mechanism at the same wind speed of 6 m/s. The response demonstrates self-sustaining, steady, nearly harmonic oscillation, which represents the fundamental properties of vortex-induced vibration^43^

#### Effect of integration of elastic and tuned mass mechanisms on bladeless wind turbine performance

**Fig. 14:**
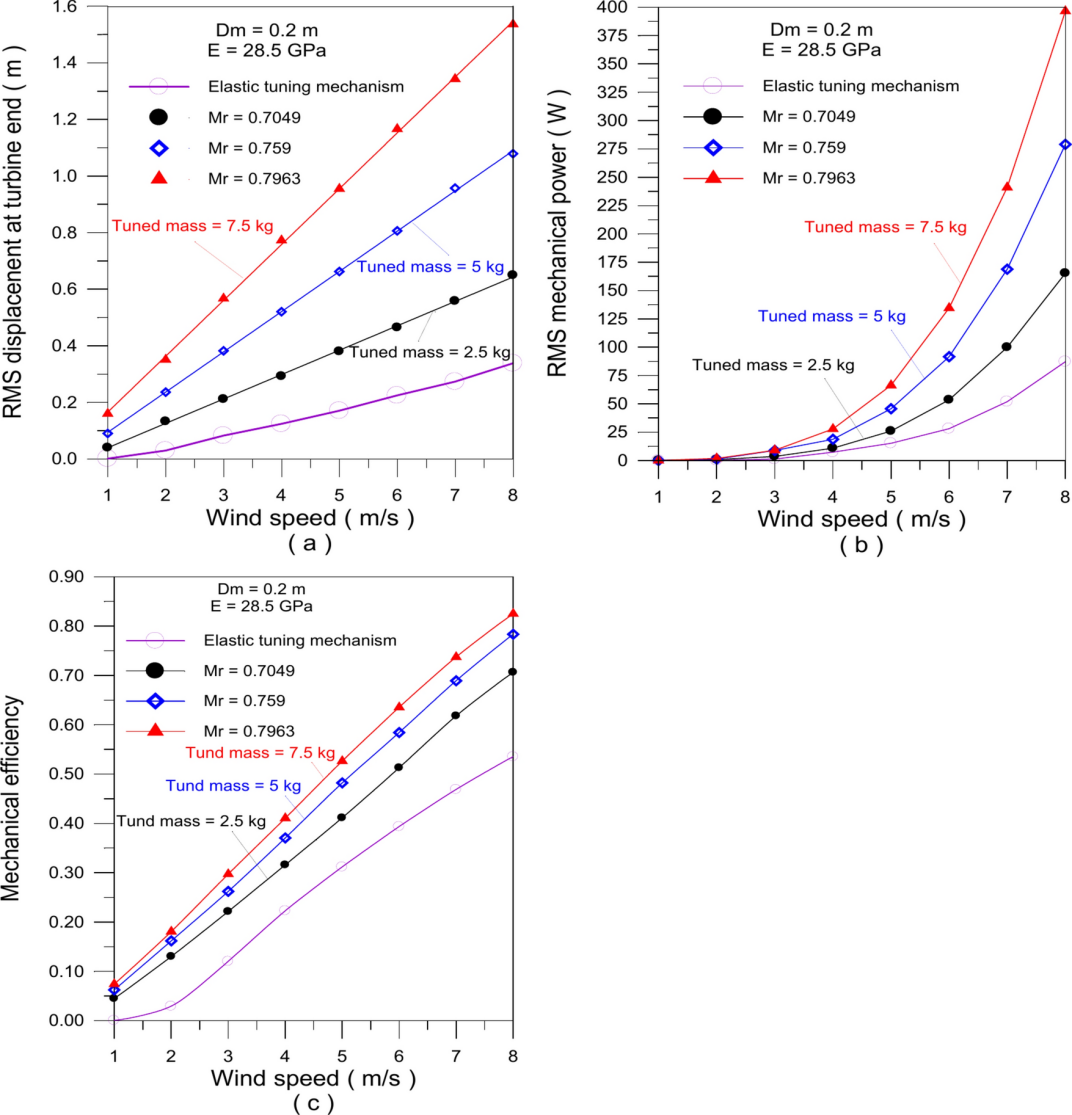
The turbine performance with and without the use of the tuned mass mechanism. (**a**) RMS displacement of mast end, (**b**) RMS mechanical power, (**c**) mechanical efficiency.

The combining between adjusting mass mechanism and the elastic tuning mechanism results in a significant improvement in the turbine’s dynamic response shown in Fig. 14, the integration of both mechanisms improves the amplitude of vibration, output mechanical power, and mechanical efficiency. Figure 14a findings show that increasing the tuned mass causes the vibration amplitude of the mast end to grow to 1.536 m with $M_{r} = 0.7963$ at $8\,m/s$ wind speed rather than 0.338 m using only the elastic tuning mechanism, As seen in Fig. 14b, using the tuned mass significantly increases the mechanical power output. It is possible to obtain 396.52 W of mechanical power at 8 m/s wind speed with a tuned mass of 7.5 kg ($M_{r} = 0.7963$), as compared to 100 W using only an elastic tuning mechanism at the same wind speed. The implementation of the tuned mass also greatly enhances mechanical efficiency, as shown in Fig. 14c. The mechanical efficiency for a tuned mass of 7.5 kg is 0.825 (55.7% increase) at 8 m/s wind speed as instead of 0.53 when applying the elastic tuning mechanism alone at the same wind speed.

Furthermore, it’s essential to keep in mind that the combined employment of the elastic tuning mechanism and the tuned mass mechanism significantly affects efficiency and output power, particularly at high wind speed values. As shown in Tables 4 and 5, using a stand material with a relatively high flexural modulus value and using a tuned mass mechanism decrease the stand effective length thus reducing the turbine’s overall size, especially at relatively low wind speed values. This combined approach not only enhances performance but also contributes to a more compact, efficient and turbine design.

**Table 5:** Stand effective length and the tuned mass position, $D_{m} = 0.2\,m$.

$V\,\,(m/s)$	$m_{t} = 2.5\,kg$		$m_{t} = 5\,kg$		$m_{t} = 7.5\,kg$	
	$L_{b\, - \,eff} \,\,(m)$	$s\,\,(m)$	$L_{b\, - \,eff} \,\,(m)$	$s\,\,(m)$	$L_{b\, - \,eff} \,\,(m)$	$s\,\,(m)$
2	2.122	4.711	1.839	4.72	1.665	4.76
3	1.472	4.611	1.264	4.62	1.138	4.675
4	1.132	4.51	0.976	4.522	0.872	4.57
5	0.922	4.432	0.792	4.442	0.714	4.441
6	0.782	4.32	0.673	4.332	0.604	4.351
7	0.676	4.241	0.582	4.24	0.528	4.25
8	0.602	4.13	0.519	4.14	0.465	4.148
9	0.539	4.05	0.469	1.03	0.422	4.05
10	0.492	3.946	0.424	3.94	0.384	3.936

## Limitations

The primary limitations are the tuning mechanisms (elastic tuning mechanism and tuned mass mechanism), increased cost, complexity, maintenance of moving parts, and fatigue damage to the stand. The current work introduces an extremely important output that demonstrates that the essential tuning relations are dependent on one input, the output of the air speed sensor. For the elastic tuning mechanism, the relation between the effective stand length and the wind speed is very smooth and can be fitted to obtain an accurate relation between $L_{b\, - \,eff}$ and V, whereas for the tuned mass mechanism, the relation between the tuned mass position s and the wind speed V has a linear relationship. These relationships lead to a straightforward control process. The elastic tuning mechanism has been constructed as a sliding sleeve supported by two antifriction guides using linear bearings to limit the amount of power required operating it, resulting in a simple design at a reasonable cost. The tuned mass mechanism can be created with a suitable motor connected to a timing belt mechanism, resulting in a lightweight and simple design with accurate motion at an acceptable cost.

It is critical to consider fatigue stress in the design of bladeless wind turbines, including the stand. Fatigue loading, generated by cyclic presses from wind forces, can have significant effects on the turbine’s long-term reliability and performance. The choice of stand material is crucial in minimizing fatigue effects. Carbon fiber composites are a great choice because of their high strength-to-weight ratio and fatigue resistance, making them extremely resistant to cyclic loads. This is especially essential in wind turbine applications, where components are subjected to frequent stress variations. In addition, carbon fiber composites are highly corrosion resistant, making them ideal for outdoor areas with harsh weather conditions. While rain-flow analysis is an excellent technique for normal bladed turbines, direct application to bladeless turbines could require significant modifications due to specific loading conditions, which are outside the scope of the current study.

## Conclusions

This study employs an advanced mathematical model to investigate the potential for maintaining optimal performance without the efficiency decline that observed in conventional BWTs, as well as being reducing the overall size of the turbine and enhancing the performance of the VIV-BWT through employing both elastic and mass-tuned mechanisms for optimal design. The following key conclusions can be drawn from the study:

- The utilization of the mast unit’s equivalent mass and equivalent polar mass moment of inertia at the free end of the cantilevered beam (stand) is crucial in the designing the turbine (including geometric parameters and material selection). This approach ensures that the turbine meets lock-in conditions within a specified range of wind speeds.
- Regardless of changing wind speed, geometric parameter, and stand material type by applying the elastic mechanism you can set the required effective length of the stand to achieve resonance at any wind speed within 2–8 m/s, with a 99.2% increase in efficiency at 7 m/s compared to conventional BWTs which experience efficiency drops post lock-in speed.
- Selecting composite materials such as carbon fiber with appropriate flexural modulus, is crucial for enhancing BWT performance. This choice improves mechanical efficiency, optimizes the structural design, and allows for precise control over turbine dimensions and stability across varying wind speeds.
- For relatively low values of the flexural modulus of the stand material, the elastic tuning mechanism alone can suffice.
- Incorporating the tuning mass mechanism becomes necessary when using higher values of the stand material’s flexural modulus. This mechanism reduces the required effective length of the stand by adjusting the adjustable mass position, thus minimizing the overall size of the turbine, especially at lower wind speeds.
- Overall, the implementation of the tuning mass mechanism in combination with the elastic tuning mechanism results in substantial enhancements in the all performance of BWTs. For instance, at a wind speed of 8 m/s, the mechanical efficiency increased by 55.7% with a tuned mass of 7.5 kg compared to applying the elastic tuning mechanism alone.

This study emphasizes the importance of applying these mechanisms that pave the way for designing more efficient and applicable BWTs to address the current operational limitations and offer a promising and more sustainable solution for vortex-induced vibration energy harvesting specially in urban areas where sustainable energy solutions are crucial. Beyond performance improvements, the study suggests that the proposed mechanisms could positively impact the lifecycle of BWTs by enhancing their durability and efficiency.

## Data Availability

All data generated or analyzed during this study are included in this published article.

## Appendix A: Natural frequencies of a cantilever beam with an end mass ($M_{L} \,\,and\,\,I_{L}$)

The governing equation of motion for undamped free vibrations of a uniform Euler–Bernoulli beam may be written as:

30 $$\frac{{\partial^{4} }}{{\partial \,x^{4} }}\left( {E\,I\,Y(x,t)} \right) + m_{s} \,\frac{{\partial^{2} Y(x,t)}}{{\partial \,t^{2} }} = 0$$

where $E$ is the Flexural modulus of elastic beam, $I$ is the second moment of area of the cross section, and $m_{s}$ is the mass per unit length of the stand (beam). The boundary conditions for the beam shown in Fig. 2 are,

31 $$\left. \begin{gathered} At\,\,x = 0\,(displacement = 0\,,\,\,slope = 0):\,\,\,Y(x,t) = 0\,\,;\,\,\frac{\partial \,Y(x,t)}{{\partial \,x}} = 0\, \hfill \\ At\,\,x = L\,,\,\,bending\,\,moment:\,\,E\,I\,\,\frac{{\partial^{2} \,Y(x,t)}}{{\partial \,x^{2} }} + I_{L} \,\frac{{\partial^{3} \,Y(x,t)}}{{\partial \,t^{2} \,\partial \,x}} = 0\,\,;\, \hfill \\ \,\,\,\,\,\,\,\,\,\,\,\,\,\,\,\,\,\,\,\,\,\,\,\,shearing\,\,force:\,\,\,\,\,\,\,E\,I\,\,\frac{{\partial^{3} \,Y(x,t)}}{{\partial \,x^{3} }} - M_{L} \,\frac{{\partial^{2} \,Y(x,t)}}{{\partial \,t^{2} \,}} = 0\hfill \\ \end{gathered} \right\}$$

For a uniform cross-section beam, Eq. (30) becomes:

32 $$\frac{{\partial^{4} Y(x,t)}}{{\partial \,x^{4} }} + \frac{{m_{s} }}{E\,I}\,\frac{{\partial^{2} Y(x,t)}}{{\partial \,t^{2} }} = 0$$

Equation (32) is the free-vibration equation of an Euler–Bernoulli beam with uniform shape. To solve Eq. (32), which is fourth-order in geographical variables and second-order in time, apply the variable separation approach as follows:

33 $$Y(x,t) = \sum {\varphi_{i} \,(x)\,y_{i} \,(t)}$$

where $\varphi_{i} \,(x)$ and $y_{i} \,(t)$ are called the mode shape and the modal response factor, respectively. If only the first mode is considered, Eq. (33) becomes,

34 $$Y\,(x,t) = \varphi \,(x)\,y\,(t)$$

Substituting Eq. (34) into Eq. (32) to give.

35 $$\frac{E\,I}{{m_{s} }}\,\frac{1}{\varphi \,(x)}\frac{{d^{4} \varphi \,(x)}}{{d\,x^{4} }} = \, - \,\frac{1}{y\,(t)}\,\frac{{d^{2} y(t)}}{{d\,t^{2} }}$$

The left-hand side of Eq. (35) depends on $x$ alone while the right-hand side depends on $t$ alone, where $x\,\,and\,\,t$ are independent. According to the typical argumentation in the method of separation of variables, the constant $\gamma$ must being the same in both sides of Eq. (35):

36 $$\frac{E\,I}{{m_{s} }}\,\frac{1}{\varphi \,(x)}\frac{{d^{4} \varphi \,(x)}}{{d\,x^{4} }} = \, - \,\frac{1}{y\,(t)}\,\frac{{d^{2} y(t)}}{{d\,t^{2} }} = \gamma$$

yielding

37 $$\,\frac{{d^{4} \varphi \,(x)}}{{d\,x^{4} }}\, - \,\gamma \,\frac{{m_{s} }}{E\,I}\,\varphi \,(x) = 0$$

38 $$\frac{{d^{2} \,y(t)}}{{d\,t^{2} }} + \gamma \,y\,(t) = 0$$

It follows from Eq. (38) that $\gamma$ is a positive constant which can be expressed as $\gamma = \omega^{2}$ and the response is oscillatory without growing or decaying.

Equations (37) and (38) can be written as follows.

39 $$\,\frac{{d^{4} \varphi \,(x)}}{{d\,x^{4} }}\, - \,\beta^{4} \,\varphi \,(x) = 0$$

40 $$\frac{{d^{2} y(t)}}{{dt^{2} }} + \omega^{2} y(t) = 0$$

where $\beta^{4} = \frac{{\omega^{2} \,m_{s} }}{E\,I}$, $EI\,\,and\,\,m_{s}$ are constants.

Let’s first solve for the space variable Eq. (39). Equation (39) is homogenous ordinary differential equation of 4th order, whose solution is given by

41 $$\varphi (x) = A\,\cos \,(\beta \,x) + B\,\cosh \,(\beta \,x) + C\,\sin (\,\beta \,x) + D\,\sinh \,(\beta \,x)$$

Equation (40) is 2nd order homogenous differential equation, its solution is given by,

42 $$y\,(t) = E_{1} \,\cos \,(\omega \,t) + E_{2} \,\sin \,(\omega \,t)$$

where *A, B, C, D, E1 and E2* are unknown constants.

Equation (34) can be employed in Eq. (31) to obtain.

43 $$\left. \begin{gathered} At\,x = 0:\,\,\,\varphi \,(x) = 0\,\,,\,\,\varphi^{\prime}\,(x) = \frac{d\,\varphi \,(x)}{{d\,x}} = 0 \hfill \\ \,At\,\,x = L\,:\,\,E\,I\,\frac{{d^{2} \varphi \,(x)}}{{d\,x^{2} }} - \omega^{2} \,I_{L} \,\frac{d\,\varphi \,(x)}{{d\,x}} = 0\,\,,\, \hfill \\ \,\,\,\,\,\,\,\,\,\,\,\,\,\,\,\,\,\,\,\,\,\,\,E\,I\,\frac{{d^{3} \varphi \,(x)}}{{d\,x^{3} }} + \omega^{2} \,M_{L} \,\varphi \,(x) = 0 \hfill \\ \end{gathered} \right\}$$

Substituting Eq. (43) into Eq. (41), at *x*=*0,* gives

44 $$\left. \begin{gathered} A + B = 0 \hfill \\ C + D = 0 \hfill \\ \end{gathered} \right\}$$

Equation (41) becomes

45 $$\varphi \,(x) = A\,\left[ {\cos \,(\beta \,x) - \cosh \,(\beta \,x)} \right] + C\,\left[ {\sin \,(\beta \,x) - \sinh \,(\beta \,x)} \right]$$

Substituting Eq. (45) into Eq. (43), at $x = L$*,* we get

46 $$\begin{gathered} - A\,\left[ {\cos \,(\beta \,L) + \cosh \,(\beta \,L) - \frac{{\omega^{2} \beta \,I_{L} }}{{E\,I\,\beta^{2} }}\,\left( {\sin \,(\beta \,L) + \sinh \,(\beta \,L)} \right)} \right] \hfill \\ \,\,\,\,\,\,\,\,\,\,\,\,\,\,\,\,\,\,\,\,\, - C\,\left[ {\sin \,(\beta \,L) + \sinh \,(\beta \,L) + \frac{{\omega^{2} \beta \,I_{L} }}{{E\,I\,\beta^{2} }}\,\left( {\cos \,(\beta \,L) - \cosh \,(\beta \,L)} \right)} \right] = 0 \hfill \\ \end{gathered}$$

47 $$\begin{gathered} A\,\left[ {\sin \,(\beta \,L) - \sinh \,(\beta \,L) + \frac{{\omega^{2} M_{L} }}{{E\,I\,\beta^{3} }}\,\left( {\cos \,(\beta \,L) - \cosh \,(\beta \,L)} \right)} \right] \hfill \\ \,\,\,\,\,\,\,\,\,\,\,\,\,\,\,\,\,\,\,\,\, + C\,\left[ { - \cos \,(\beta \,L) - \cosh \,(\beta \,L) + \frac{{\omega^{2} M_{L} }}{{E\,I\,\beta^{3} }}\,\left( {\sin \,(\beta \,L) - \sinh \,(\beta \,L)} \right)} \right] = 0 \hfill \\ \end{gathered}$$

After defining $\lambda = \beta \,L$, rewrite Eqs. (46) and (47) as follows:

48 $$\left[ {\begin{array}{*{20}c} {\cos \,(\lambda ) + \cosh \,(\lambda ) - \left( {\frac{{\lambda^{3} I_{L} }}{{m_{s} \,L^{3} }}} \right)\,\left( {\sin \,(\lambda ) + \sinh \,(\lambda )} \right)} & {\,\sin \,(\lambda ) + \sinh \,(\lambda ) + \left( {\frac{{\lambda^{3} I_{L} }}{{m_{s} L^{3} }}} \right)\,\left( {\cos \,(\lambda ) - \cosh \,(\lambda )} \right)} \\ {\sin \,(\lambda ) - \sinh \,(\lambda ) + \left( {\frac{{\lambda \,M_{L} }}{{m_{s} L}}} \right)\,\left( {\cos \,(\lambda ) - \cosh \,(\lambda )} \right)} & {\,\,\,\, - \cos \,(\lambda ) - \cosh \,(\lambda ) + \left( {\frac{{\lambda \,M_{L} }}{{m_{s} L}}} \right)\,\left( {\sin \,(\lambda ) - \sinh \,(\lambda )} \right)} \\ \end{array} } \right]\,\left[ {\begin{array}{*{20}c} A \\ C \\ \end{array} } \right] = \left[ {\begin{array}{*{20}c} 0 \\ 0 \\ \end{array} } \right]$$

Setting the determinant of the above coefficient matrix gives the characteristic equation of the differential eigenvalue problem as

49 $$\begin{gathered} 1 + \cos \,(\lambda )\,\cosh \,(\lambda ) - \lambda \,\frac{{M_{L} }}{{m_{s} L}}\,\left( {2\,\cos \,(\lambda )\,\sinh \,(\lambda ) + \sin \,(\lambda )\,\cosh \,(\lambda )} \right) \hfill \\ \,\,\,\,\,\,\,\, - \frac{{\lambda^{3} \,I_{L} }}{{m_{s} \,L^{3} }}\,\left( {\cosh \,(\lambda )\,\sin \,(\lambda ) + \sinh \,(\lambda )\,\cos \,(\lambda )} \right)\, + \frac{{\lambda^{4} \,M_{L} I_{L} }}{{m_{s}^{2} \,L^{4} }}\,\left( {1 - \cos \,(\lambda )\,\cosh \,(\lambda )} \right) = 0 \hfill \\ \end{gathered}$$

## Appendix B: Orthogonality of the eigenfunctions

Substituting two distinct solutions (modes $r_{1} \,\,and\,\,r_{2}$) of the eigenvalue problem into the spatial Eq. (39) gives.

50 $$EI\frac{{d^{4} \phi_{r1} (x)}}{{dx^{4} }} = \omega_{r1}^{2} m_{s} \phi_{r1} (x)$$

51 $$EI\frac{{d^{4} \varphi_{{r_{2} }} (x)}}{{d\,x^{4} }} = \omega_{{r_{2} }}^{2} \,m_{s} \,\varphi_{{r_{2} }} (x)$$

Multiplying equation (50) by $\varphi_{{r_{2} }} (x)$ and integrating over the beam length gives

52 $$\int\limits_{0}^{{L_{b} }} {\varphi_{{r_{2} }} (x)\,EI\frac{{d^{4} \varphi_{{r_{1} }} (x)}}{{d\,x^{4} }}\,dx = \omega_{{r_{1} }}^{2} \,\int\limits_{0}^{Lb} {\varphi_{{r_{2} }} (x)\,m_{s} \,\varphi_{{r_{1} }} (x)} \,dx\,\,}$$

Applying integration by parts twice to the left hand side of Eq. (52) and using Eq. (15), one gets

53 $$\begin{gathered} \int\limits_{0}^{{L_{b} }} {\frac{{d^{2} \varphi_{{r_{2} }} (x)}}{{d\,x^{2} }}\,EI\frac{{d^{2} \varphi_{{r_{1} }} (x)}}{{d\,x^{4} }}\,dx} = \hfill \\ \,\,\,\,\,\,\,\,\,\,\,\,\,\,\,\omega_{{r_{1} }}^{2} \,\left\{ {\int\limits_{0}^{Lb} {\varphi_{{r_{2} }} (x)\,m_{s} \,\varphi_{{r_{1} }} (x)} \,dx\, + \varphi_{{r_{2} }} (L_{b} )\,M_{L} \,\varphi_{{r_{1} }} (L_{b} ) + \left( {\frac{{d\varphi_{{r_{2} }} (x)}}{d\,x}\,I_{L} \,\frac{{d\varphi_{{r_{1} }} (x)}}{d\,x}} \right)_{{x = L_{b} }} \,\,\,\,\,} \right\} \hfill \\ \end{gathered}$$

Multiplying equation (51) by $\varphi_{{r_{1} }} (x)$ and following a similar procedure gives

54 $$\begin{gathered} \int\limits_{0}^{{L_{b} }} {\frac{{d^{2} \varphi_{{r_{2} }} (x)}}{{d\,x^{2} }}\,EI\frac{{d^{2} \varphi_{{r_{1} }} (x)}}{{d\,x^{2} }}\,dx} = \hfill \\ \,\,\,\,\,\,\,\,\,\,\,\,\,\,\,\,\,\omega_{{r_{2} }}^{2} \,\left\{ {\int\limits_{0}^{Lb} {\varphi_{{r_{2} }} (x)\,m_{s} \,\varphi_{{r_{1} }} (x)} \,dx\, + \varphi_{{r_{2} }} (L_{b} )\,M_{L} \,\varphi_{{r_{1} }} (L_{b} ) + \left( {\frac{{d\varphi_{{r_{2} }} (x)}}{d\,x}\,I_{L} \,\frac{{d\varphi_{{r_{1} }} (x)}}{d\,x}} \right)_{{x = L_{b} }} \,\,\,} \right\} \hfill \\ \end{gathered}$$

Subtracting equation (54) from (53) and recalling that $r_{1} \,\,and\,\,r_{2}$ are distinct modes $(\omega_{{r_{1} }}^{2} \ne \omega_{{r_{2} }}^{2} )$, the orthogonally condition of the eigenfunctions is

55 $$\begin{gathered} (\omega_{{r_{1} }}^{2} - \omega_{{r_{2} }}^{2} )\int\limits_{0}^{Lb} {\varphi_{{r_{2} }} (x)\,m_{s} \,\varphi_{{r_{1} }} (x)} \,dx\, + \varphi_{{r_{2} }} (L_{b} )\,M_{L} \,\varphi_{{r_{1} }} (L_{b} ) + \left( {\frac{{d\varphi_{{r_{2} }} (x)}}{d\,x}\,I_{L} \,\frac{{d\varphi_{{r_{1} }} (x)}}{d\,x}} \right)_{{x = L_{b} }} = 0 \hfill \\ where\,\,\,\,\,\omega_{{r_{1} }}^{2} \ne \omega_{{r_{2} }}^{2} \hfill \\ \end{gathered}$$

Normalization of the eigenfunctions:

It follows from the orthogonality condition given by equation (55) that

56 $$\int\limits_{0}^{Lb} {\varphi_{{r_{2} }} (x)\,m\,\varphi_{{r_{1} }} (x)} \,dx\, + \varphi_{{r_{2} }} (L_{b} )\,M_{L} \,\varphi_{{r_{1} }} (L_{b} ) + \left( {\frac{{d\varphi_{{r_{2} }} (x)}}{d\,x}\,I_{L} \,\frac{{d\varphi_{{r_{1} }} (x)}}{d\,x}} \right)_{{x = L_{b} }} = \,\,\,\delta_{r\,s}$$

where $\delta_{r\,s}$ is the Kronecker delta. The Kronecker delta function is equal to 1 when the indices $r_{1} \,\,and\,\,r_{2}$ are equal, while yields a 0 value when the indices $r_{1} \,\,and\,\,r_{2}$ are unequal. Hence, $r_{1} = r_{2}$ can be used to solve for the modal amplitude $A_{r}$ of the eigenfunctions (i.e., to normalize the eigenfunctions). The eigenfunctions normalized according to equation (56) are called the mass-normalized eigenfunctions.

$A_{r}$ can be obtained as,

57 $$A_{r} = \frac{1}{{\sqrt {\int_{0}^{{L_{b} }} {m_{s} \phi^{*2} (x)dx + \phi^{*2} (L_{b} )M_{L} + \phi^{*^{\prime}2} (L_{b} )I_{L} } } }},\,\,\,\,\,\phi_{r} (x) = A_{r} \phi^{*} (x)$$

## List of symbols

$$A_{s}$$: Swept area, m^2^
$C_{L} (x,t)$: The oscillating lift coefficient
$C_{Lo}$: The amplitude of the fluctuating lift coefficient on a stationary cylinder
$D_{e}$: The dissipative energy, Nm
$$D_{m}$$: Mast diameter, m
E: The flexural modulus of the elastic stand, Pa
F: An empirical parameter
$F_{L}$: The y-direction lift force, N
G: An empirical parameter
I: The second moment of area of the cross-section of the stand, m^4^
$I_{L}$: Mass moment of inertia of the mast unit about z-axis at the tip of the stand, kg m^2^
$I_{m}$: Mass moment of inertia of the mast about z-axis at the center of the mast, kg m^2^
$I_{rod}$: Mass moment of inertia of the guide rod about z-axis at the center of the guide, kg m^2^
$I_{t}$: Mass moment of inertia of the tunable mass about z-axis at the center of mass, kg m^2^
*L*: Cantilever beam length, m
$L_{b}$: Working length of the stand, m
$L_{rod}$: Guide rod length, m
$L_{b\,\, - \,\,eff}$: Stand effective length, m
$\overline{L}_{m}$: The distance between the tip of the stand and the center of the mast, m
$\overline{L}_{rod}$: The distance between the tip of the stand and the center of the guide rod, m
$m_{m}$: The mass of the mast, kg
$m_{rod}$: The mass of the guide rod, kg
$m_{t}$: The tunable mass, kg
$m_{s}$: The mass per unit length of the stand, kg/m
M: The aerodynamic moment exerted from the mast on the tip of the stand, Nm
$M_{L}$: Equivalent mass of the mast unit at the tip of the stand, kg
$P_{f}$: The available fluid power, W
$P_{m}$: Power transmitted to the BWT, W
$Q(x,t)$: A dimensionless weak variable that describes the oscillating dynamics
$$R_{e}$$: Reynolds number, $$300\, < R_{e} = \frac{{\rho \,V\,D_{m} }}{\mu }\,\, < 3 \times 10^{5} \,\,.$$
s: The distance between the tip of the stand and the center of the tunable mass, m
$$S_{t}$$: Strouhal number, $$S_{t} = 0.21.$$
t: Time, s
T: The kinetic energy. Nm
U: The potential energy, Nm
V: Air velocity, $$2 \le V \le 10\,m/s\,.$$
W: The non-conservative work of aerodynamic force, Nm
$y_{i} (t)$: The modal response factor
$Y(x,t)$: Deflection of the stand at position x and time t, m
$Y(L_{b} ,t)$: The free end displacement of the stand
$Y^{\prime}(L_{b} ,t)$: The free rotation of the stand
x,y,z: Cartesian coordinates

## Greek symbols

$\alpha$: An empirical parameter
$\gamma$: The ratio of rotation to displacement at the stand end
$\eta_{m}$: The mechanical efficiency, $\eta_{m} = {{P_{m} } \mathord{\left/ {\vphantom {{P_{m} } {P_{f} }}} \right. \kern-0pt} {P_{f} }}\,.$
$\theta (L_{b} ,t)$: The rotation angle at the tip of the stand
$$\lambda$$: The eigenvalue
$\lambda_{r}$: The eigenvalue for $r^{th}$ mode of vibration
$\mu$: The absolute viscosity of air, $\mu = 1.525 \times 10^{ - \,5} \,Pa\,s\,\,at\,\,20\,\,{}^{o}C$
$$\rho$$: Air density, $$\rho = 1.225\,{{\,\,\,kg} \mathord{\left/ {\vphantom {{\,\,\,kg} {m^{3} }}} \right. \kern-0pt} {m^{3} }}$$
$\varphi_{i} (x)$: The mode shape (eigenfunction)
$\varphi_{r} (x)$: Eigenfunction for $r^{th}$ mode of vibration
$\omega_{r}$: The undamped natural frequency (eigen frequency for $r^{th}$ mode of vibration), rad/s
$\omega_{s}$: Vortex shedding angular frequency, $\omega_{s} = 2\rho S_{t} V/D_{m}$

## Abbreviations

VIV: Vortex-induced vibration
BWT: Bladeless wind turbine
MPC: Model predictive control
RMS: Root mean square
PTO: Power take-off
